# Response Monitoring Theta-Band Activities Across Emotional Contexts in Schizophrenia and Bipolar Spectrum Disorders

**DOI:** 10.1016/j.bpsgos.2025.100540

**Published:** 2025-05-27

**Authors:** Takakuni Suzuki, Margo W. Menkes, Melvin G. McInnis, Jian Kang, Tara A. Niendam, Maureen A. Walton, Patricia J. Deldin, Ivy F. Tso, Stephan F. Taylor

**Affiliations:** a Department of Psychology, University of Tulsa, Tulsa, Oklahoma; b Department of Psychiatry, University of Michigan, Ann Arbor, Michigan; c Department of Psychology, University of Michigan, Ann Arbor, Michigan; d Department of Psychiatry, University of Maryland School of Medicine, College Park, Maryland; e Department of Biostatistics, University of Michigan, Ann Arbor, Michigan; f Department of Psychiatry, University of California Davis, Davis, California; g Department of Psychiatry & Behavioral Health, The Ohio State University, Columbus, Ohio

**Keywords:** Emotion appraisal, Event-related potential, Executive function, Neural oscillation, Time-frequency decomposition

## Abstract

**Background:**

Schizophrenia spectrum disorder (SZ) and bipolar spectrum disorder (BD) have traditionally been treated as different conditions but share many characteristics, including cognitive control deficits. Electroencephalogram (EEG) indicators of response monitoring, including error-related negativity (ERN) and theta-band activities (4–8 Hz), have been proposed as transdiagnostic indicators of cognitive control. Research has found that the ERN and theta power are blunted in SZ, but findings have been less consistent in BD. Individuals with SZ and BD also show difficulty in emotional contexts. However, no research has investigated response-monitoring theta activities in SZ and BD concurrently or in emotional contexts.

**Methods:**

Data were collected from 32 participants with SZ, 33 participants with BD, and 33 healthy control (HC) participants. EEG was recorded while participants completed 3 modified flanker tasks using arrow, unpleasant, and pleasant stimuli. Effects of group and task on postresponse event-related potentials (ERN, correct-related negativity), theta total power, and theta intertrial phase coherence (ITPC) were investigated using mixed analysis of covariance, controlling for age and accuracy.

**Results:**

The SZ group did not show the ERN modulation by task that was found in the HC and BD groups. The SZ group showed attenuated theta power across all tasks, and the BD group showed attenuated power only on error trials with unpleasant stimuli. Both SZ and BD groups showed emotional modulation for theta ITPC. Theta power was correlated across tasks, suggesting that it is task invariant, while ITPC was not, suggesting that it is task-specific.

**Conclusions:**

SZ and BD show different effects of emotional stimuli on cognitive control. To elucidate similarities and differences, concurrent data collection from individuals with SZ and BD across contexts is needed.

## Plain Language Summary

Traditionally, schizophrenia (SZ) and bipolar disorder (BD) have been considered to reflect distinct experiences and difficulties. However, research suggests that people with SZ and BD share many experiences, including difficulty adapting to emotional situations. The goal of this project was to understand the brain mechanisms that underlie such difficulties in people with SZ and BD by measuring brain activities at the scalp (electroencephalogram) while they made decisions about pictures with emotional content. The findings suggest that people with SZ showed reduced brain activities that may underlie such difficulty adapting, and people with BD showed similar reduction but to a lesser extent.

Schizophrenia spectrum disorder (SZ) and bipolar spectrum disorder (BD) are serious and chronic psychiatric conditions that affect approximately 2% of the population and are associated with significant health difficulties and functional impairments (1, 2, 3, 4, 5). SZ and BD have traditionally been treated as distinct categorical disorders (6). While some differences have been reported (e.g., developmental, emotional processing) (7, 8, 9, 10), research indicates that these disorders share several characteristics (e.g., biological characteristics, genetic risks) (11, 12, 13) and are better conceptualized as variants on the same spectrum. However, the relatively few concurrent investigations of SZ and BD prohibit clear delineation of the similarities and differences.

Cognitive deficits are ubiquitous and could shed light on the relationship between SZ and BD (14). In individuals with SZ and BD, cognitive deficits are present by the first episode (15) and are associated with functional impairments (16). Furthermore, current pharmacotherapy has little effect on cognitive deficits (17), and while psychosocial treatments and vocational rehabilitation show promise, many patients continue to experience some functional impairment (18,19). Thus, understanding the underlying neural mechanisms of cognitive deficits in SZ and BD are imperative for improving and developing effective interventions.

Response monitoring is one form of cognitive control with strong experimental support. Specifically, the error-related negativity (ERN) (20,21) is an event-related potential (ERP) characterized by a more negative deflection in the electroencephalogram (EEG) within 100 ms after making an erroneous response compared to correct responses (correct-related negativity [CRN]). It is thought to reflect an alerting system of monitoring response outcomes to adjust future behaviors and to originate from the anterior cingulate cortex and supplementary motor area (22). The ERN has been identified as a transdiagnostic biomarker for various disorders (23) and is a promising neural activity indicator to elucidate the mechanism that underlies cognitive deficits among individuals with SZ and BD. Specifically, research has consistently shown that the ERN is blunted in individuals with SZ and substance use disorders but abnormally larger in individuals with obsessive-compulsive disorder (OCD) (24,25). The ERN may be blunted among individuals with BD (26), although this literature is smaller and less consistent (27,28).

Individuals with SZ and BD also exhibit difficulties with emotion regulation and experience excessively heightened negative and positive affective states (29, 30, 31, 32, 33). Emotional states can compromise the ability to effectively engage cognitive control, such as resulting in making rash decisions (e.g., negative and positive urgency) (34,35). For example, in SZ, reduced emotional information processing has been related to social challenges (36). Thus, examining the recruitment of cognitive control in emotional contexts may shed light on specific difficulties in individuals with SZ and BD. Research has shown that, while task and context modulate the ERN (e.g., instructions that emphasize high accuracy increase the ERN amplitude) (37,38), individuals with SZ show such modulations to a lesser extent (38). This could reflect that individuals with SZ have difficulty adjusting cognitive control engagement across contexts, such as emotional contexts, in addition to the general cognitive difficulty. Several studies that investigated information-processing ERPs (e.g., late positive potential) suggest that individuals with SZ have difficulty recruiting cognitive resources in emotional contexts, and individuals with BD show a similar reduction although to a lesser extent (8,39). To our knowledge, there is no previous work differentiating cognitive processes across emotional contexts among individuals with SZ and BD using a response-monitoring paradigm, which reflects an active engagement with the task and is proximal to behavior. Flanker tasks using emotional images from the International Affective Picture System (IAPS) (40) elicit larger ERNs than neutral images in undergraduate participants (41), creating an opportunity to examine the emotional modulation of response monitoring. This paradigm allows us to investigate the recruitment of cognitive control in general as well as the way it is modulated by emotional appraisal in SZ and BD.

While the ERN has a rich literature, it only reflects a summary of all underlying neural oscillatory activities. Specifically, the ERN is primarily due to increased theta-band (4–8 Hz) power (i.e., amplitude^2^) and phase resetting (quantified as intertrial phase coherence [ITPC]) when making an error (42, 43, 44). Furthermore, theta activities underlie other ERP components and have been proposed as a unifying transdiagnostic measure to consolidate the broad ERP literature (45). Thus, identifying the time-frequency activities that underlie response monitoring could shed light on transdiagnostic neural mechanisms of cognitive deficits in SZ and BD. Consistent with the ERN literature, individuals with SZ and BD show reduced theta power and ITPC reflecting other aspects of cognitive control (e.g., Go/NoGo task stimulus-locked activities) (46, 47, 48, 49). Error-related theta power abnormalities have been identified in individuals with SZ (50,51), although phase abnormalities are less understood, and neither has been investigated among individuals with BD.

This study had 2 broad aims. The first aim was to investigate cognitive control processes, primarily operationalized as response-monitoring theta activities (power, ITPC), among individuals with SZ or BD. We hypothesized that participants with SZ or BD would exhibit reduced theta power and ITPC relative to healthy control (HC) participants, with this reduction being to a greater degree in SZ. The second aim was to investigate the effects of emotional stimuli appraisal on response monitoring among individuals with SZ or BD using IAPS unpleasant and pleasant images to modify flanker tasks (40). These tasks were administered in addition to the arrow flanker task to examine the effects of emotional images on cognitive control. We hypothesized that theta power and ITPC would be larger with emotional images in HC participants than arrow, reflecting additional cognitive resource recruitment. We hypothesized that participants with SZ or BD would have difficulty recruiting additional cognitive resources in emotional contexts and not show this emotion modulation. This project was preregistered as part of a larger project, and this article corresponds to part of aim 1.1 (https://osf.io/75nhw). Hypotheses for the ERN were not specifically stated in the preregistration, but theta hypotheses were based on expected ERN results, and the same pattern was hypothesized.

## Methods and Materials

### Participants

A total of 98 adults (ages 18–65 years) completed the study with usable data (33 HC, 33 BD, 32 SZ). Participants were recruited so as to match all groups on age and sex. Diagnostic groups were confirmed using the Structured Clinical Interview for DSM-5, Research Version (52). Current symptom assessments included the clinician-rated 17-item Hamilton Rating Scale for Depression (53), Young Mania Rating Scale (54), and Positive and Negative Syndrome Scale (55). See the Supplement for details. The study was approved by the University of Michigan Medical School Institutional Review Board (HUM00193488). All participants provided signed informed consent and received payment as an incentive for participation.

### EEG Tasks

#### Arrow Flanker Task

The modified Eriksen flanker task (56) is commonly used to investigate response monitoring (23,57) (Figure S1). Five arrows appeared in the center of a computer screen. There were equiprobable right and left target arrows and congruent (e.g., >>>>>) and incongruent (e.g., <<><<) conditions. Participants were instructed to click the mouse button that corresponded to the direction of the center arrow as quickly as possible, ignoring adjacent arrows. Stimuli presentation time was initially set at 200 ms. After each block, if accuracy was above 90%, feedback was provided to respond more quickly, and stimuli presentation was shortened by 20 ms in the subsequent block. If accuracy was below 75%, feedback was provided to respond more accurately, and stimuli presentation was lengthened by 20 ms in the subsequent block. Presentation time was adjusted to maximize the probability of participants making sufficient errors to be analyzable (at least 8) and to reduce accuracy-related confounds (58). Participants completed a practice block to ensure understanding and 10 test blocks of 30 trials (300 trials in total).

#### Unpleasant Flanker Task

Images from the IAPS (40) were used as stimuli instead of arrows (Figure S1). Three images appeared in the center of a computer screen, and participants were instructed to appraise and identify the valence of the center image, ignoring the adjacent images, as quickly as possible. There were equiprobable negative valence (e.g., bodily injury, aggressive animals) and neutral target (e.g., common household items) images and congruent (e.g., unpleasant-unpleasant-unpleasant) and incongruent (e.g., neutral-unpleasant-neutral) conditions. Stimuli presentation time was initially set for 400 ms, and presentation time adjustment was in 40-ms increments. The remaining parameters were identical to the arrow flanker task. See the Supplement for more information.

#### Pleasant Flanker Task

Task parameters were the same as the unpleasant flanker task, except positive valence images (cute animals, erotic pictures) were used.

### Time-Frequency Decomposition

Following preprocessing (see the Supplement), the independent component analysis-cleaned continuous EEG data were segmented into longer epochs (−3000 to 2500 ms) around retained responses. Correct trials with the closest reaction times (RTs) to error trials were selected for inclusion in time-frequency decomposition, thus including the same numbers of correct and error trials with the closest RT possible to compare trials with as similar underlying processes as possible (e.g., time to process stimuli) (42,59). Morlet wavelet convolution was conducted from 2- to 128-Hz frequencies (43 cycles), ranging from 3 cycles to 10 cycles on a logarithmically spaced scale. Power across the response-locked epoch was standardized in decibels to capture changes relative to the prestimulus baseline (−300 to −50 ms).

Collapsing all groups (HC, BD, SZ) and tasks (arrow, unpleasant, pleasant), differences in postresponse total power between error and correct trials were calculated for all electrodes to identify the maximal electrode, time, and frequency. Difference was maximal at Cz, 120 ms postresponse, and at 5.38 Hz. Windows to extract and calculate mean theta power and ITPC were created with ±100 ms and ±2 Hz on both sides of the maximal point at Cz (20–220 ms and 3.38–7.38 Hz) (Figures S2 and S3).

### Event-Related Potentials

**Figure 1:**
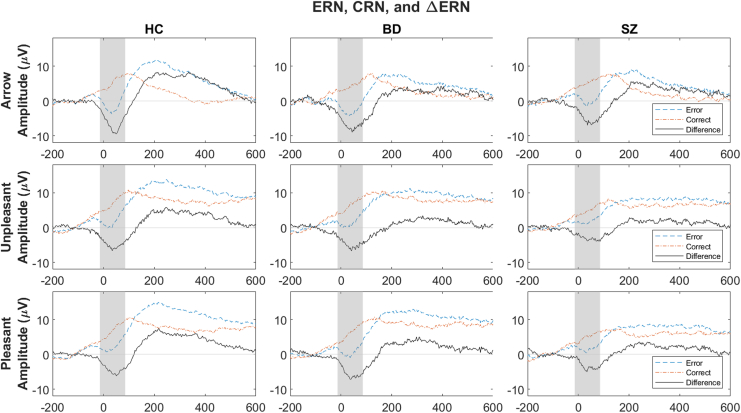
Event-related potential (ERP) plots at Cz electrode. Rows: top, arrow task; middle, unpleasant task; bottom, pleasant task. Columns: left, healthy control (HC) group; middle, bipolar spectrum disorder (BD) group; right, schizophrenia spectrum disorder (SZ) group. Blue dashed lines, error responses; orange dashed-dotted lines, correct responses; black solid line, difference between error and correct responses (error minus correct responses). The horizontal axis reflects time (−200 to 600 ms relative to response; 0 ms indicates when response was made), and the vertical axis represents voltage (μV). The gray area represents the area that ERP data were extracted from (−14 to 86 ms). Response main effects (larger error-related negativity [ERN] than correct-related negativity [CRN]) were found in all analyses (Tables 1 and 2). In analyses of covariance (ANCOVAs) testing task effects within each group, in the HC and BD groups, ERPs (ERN and CRN) were larger (more negative) in the arrow task than in the unpleasant and pleasant tasks, while ERPs were comparable across all tasks in SZ (Table 1 and Table S5). In ANCOVAs testing group effects within each task, there were no group differences in ERPs (ERN or CRN) (Table 2). Furthermore, there were no interaction effects involving response, suggesting that ΔERN (ERN minus CRN) was comparable across all groups and tasks. Line plots summarizing the findings are presented in Figure 2 (top row). Topography is presented in Figure S8.

The matched correct and error trials used in time-frequency decomposition were re-referenced to the average of TP9 and TP10 electrodes (closest to mastoid). The maximal amplitude difference between error and correct trial waveforms at Cz was 36 ms. Windows to extract and calculate mean ERPs were created with ±50 ms on both sides of the maximal points at Cz (−14 to 86 ms) (Figure 1). ERPs were also extracted using all correct trials, consistent with the traditional approach (reported in the Supplement). Matched trials make the ERP and theta activity more comparable and were chosen for main analyses and interpretation.

### Statistical Analyses

All statistical analyses were conducted in R (version 4.4.1) (60). See the Supplement for the packages used. Differences in demographic characteristics (age, sex, race) between diagnostic groups (HC, BD, SZ) were tested using 1-way analyses of variance (ANOVAs) and χ^2^ tests. Group differences in accuracy and RT were investigated using 1-way ANOVAs. To explore effects of common psychiatric medications for BD and SZ treatment, Welch’s *t* tests were used to compare EEG indicators between patients taking and not taking the given medication within the clinical sample.

Analyses of covariance (ANCOVAs), controlling for age and accuracy, were conducted to test the hypotheses and investigate group and task (emotional appraisal) effects for each EEG indicator separately (ERPs, theta power, theta ITPC). First, to test the preregistered primary hypotheses, 2-way 3 × 2 ANCOVAs were conducted to test the group effects within each task (e.g., group × response in the arrow task) and the task effects within each group (e.g., task × response in HC). In addition to planned contrasts (e.g., HC compared with SZ and BD combined), results with group, task, or interaction effects were followed to probe the source. Subsequently, to test for group × task interaction effects, a 3-way 3 × 3 × 2 ANCOVA (task [arrow, unpleasant, pleasant] × group [SZ, BD, HC] × response [correct, error]) model was tested.

As exploratory analyses, EEG measures were correlated with each other as well as clinical and behavioral measures within each group. Cluster analysis was conducted using theta-band measures to explore diagnosis-agnostic dimensions (reported in the Supplement).

For all analyses, error and correct EEG measures were extracted separately and included as separate variables (e.g., ERN, CRN) to allow testing for interaction effects (e.g., ΔERN) and main effects if no such interaction effects emerged. When no interaction effect involving the response emerged, indicating that error and correct trials had similar patterns, the main effects were reported, and response-neutral terms were used (e.g., ERP). Raw *p* values are reported for all analyses. To balance multiple comparisons (5 comparisons within each ANCOVA; type I error) and statistical power given the relatively small sample size (type II error), *p* < .01 was used as the threshold for significance. For correlational analyses, effect sizes of *r* > |0.45| (corresponds to *p* < .01 for a sample of 32) were used as the minimum threshold and consistency across the groups to guide interpretations.

## Results

Participant demographic and clinical information is presented in Table S2. Matching on age and sex across groups was successful. Race, ethnicity, education, and parental education did not differ statistically significantly across groups. Clinical measures had a pattern that would generally be expected: Participants with SZ and BD were higher on all clinical measures than HC participants, and participants with SZ had more severe psychosis symptoms than participants with BD when differences emerged. Reported prescription medication usage did not differ. Descriptive statistics for EEG and behavioral measures are presented in the Supplement (Tables S3 and S4).

### Task (Emotional Appraisal) Effects Within Each Group

**Figure 2:**
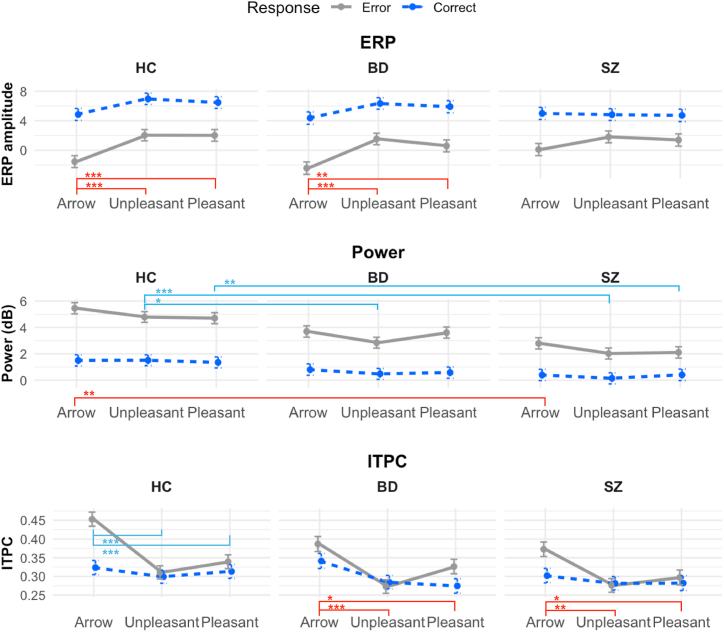
Line plots using estimated marginal means from the 3-way analysis of covariance (ANCOVA) (response × group × task) model for each electroencephalogram measure, controlling for age and accuracy rate, are depicted (Table S7). Red brackets and asterisks (above task labels) indicate statistically significant main effects based on the 2-way ANCOVA results (Tables 1 and 2). Teal brackets and asterisks (in top half of power and intertrial phase coherence [ITPC] plots) indicate the statistically significant differences by groups or by task for specific responses (in all cases, error trials) based on the 2-way ANCOVA interaction effects (Tables S5 and S6). Columns: left, healthy control (HC) group; middle, bipolar spectrum disorder (BD) group; right, schizophrenia spectrum disorder (SZ) group. ∗*p* < .01, ∗∗*p* < .001, ∗∗∗*p* < .0001. ERP, event-related potential.

The primary results are summarized in Figure 2. In 2-way ANCOVAs within each group, there were main effects of task on ERPs (no task × response interaction effect) (Table 1 and Table S5) such that they were larger in the arrow task than in the unpleasant or pleasant task in the HC (*F*_2,155.10_ = 12.47, *p* < .0001, η^2^ = 0.14) and BD (*F*_2,145.18_ = 10.66, *p* < .0001, η^2^ = 0.13) groups, while ERPs were comparable across all tasks in the SZ group (*F*_2,136.61_ = 0.81, *p* = .4461, η^2^ = 0.01). Theta power did not differ across tasks in any groups (all *F*s ≤ 2.42, *p*s ≥ .092, η^2^ ≤ 0.03). There was a main effect of task on ITPC in the BD and SZ groups such that they were larger in the arrow task than the unpleasant or pleasant tasks (all *F*s ≥ 6.34, *p*s ≤ .002, η^2^ ≥ 0.08). There was a task × response interaction effect in the HC group such that the ERN in the arrow task was larger than the ERN in other tasks and the CRN (*F*_2,145.91_ = 6.19, *p* = .0026, η^2^ = 0.08). Furthermore, response effects were present in all analyses, except for ITPC in the BD and SZ groups.

**Table 1:** Two-Way (Task × Response) Analyses of Covariance Results Within Each Group

Group	EEG Outcome	Predictor	SS	MS	*F*	*p*	η_p_^2^
HC	ERP	Age	0.52	0.52	*F*_1,35.03_ = 0.05	.8187	0.00
		Accuracy	0.15	0.15	*F*_1,157.43_ = 0.02	.9021	0.00
		Response	1266.42	1266.42	*F*_1,145.45_ = 130.74	<.0001∗∗∗	0.47
		Task	241.63	120.82	*F*_2,155.10_ = 12.47	<.0001∗∗∗	0.14
		Response × task	29.99	14.99	*F*_2,145.45_ = 1.55	.2162	0.02
	Theta power	Age	0.25	0.25	*F*_1,34.84_ = 0.16	.6950	0.00
		Accuracy	0.39	0.39	*F*_1,155.96_ = 0.25	.6188	0.00
		Response	571.95	571.95	*F*_1,145.24_ = 360.32	<.0001∗∗∗	0.71
		Task	6.26	3.13	*F*_2,155.04_ = 1.97	.1428	0.02
		Response × task	4.13	2.06	*F*_2,145.24_ = 1.30	.2757	0.02
	Theta ITPC	Age	0.03	0.03	*F*_1,34.35_ = 3.15	.0849	0.08
		Accuracy	0.14	0.14	*F*_1,61.97_ = 13.92	.0004∗∗	0.18
		Response	0.14	0.14	*F*_1,145.91_ = 14.23	.0002∗∗	0.09
		Task	0.20	0.10	*F*_2,156.56_ = 10.17	<.0001∗∗∗	0.12
		Response × task	0.12	0.06	*F*_2,145.91_ = 6.19	.0026∗	0.08
BD	ERP	Age	0.00	0.00	*F*_1,30.63_ = 0.00	.9916	0.00
		Accuracy	13.82	13.82	*F*_1,105.71_ = 1.20	.2751	0.01
		Response	1305.09	1305.09	*F*_1,128.86_ = 113.69	<.0001∗∗∗	0.47
		Task	244.80	122.40	*F*_2,145.18_ = 10.66	<.0001∗∗∗	0.13
		Response × task	28.22	14.11	*F*_2,128.86_ = 1.23	.2959	0.02
	Theta power	Age	1.70	1.70	*F*_1,30.78_ = 1.07	.3100	0.03
		Accuracy	0.53	0.53	*F*_1,156.41_ = 0.33	.5649	0.00
		Response	314.81	314.81	*F*_2,127.61_ = 197.87	<.0001∗∗∗	0.61
		Task	7.71	3.85	*F*_2,138.54_ = 2.42	.092	0.03
		Response × task	3.63	1.81	*F*_2,127.61_ = 1.14	.3231	0.02
	Theta ITPC	Age	0.00	0.00	*F*_1,27.50_ = 0.00	.9903	0.00
		Accuracy	0.13	0.13	*F*_1,65.94_ = 13.34	.0005∗∗	0.17
		Response	0.03	0.03	*F*_1,128.19_ = 3.38	.0685	0.03
		Task	0.20	0.10	*F*_2,145.25_ = 10.02	<.0001∗∗∗	0.12
		Response × task	0.04	0.02	*F*_2,128.19_ = 1.81	.1684	0.03
SZ	ERP	Age	10.58	10.58	*F*_1,30.88_ = 1.57	.2202	0.05
		Accuracy	0.10	0.10	*F*_1,154.36_ = 0.01	.9046	0.00
		Response	586.89	586.89	*F*_1,130.54_ = 86.89	<.0001∗∗∗	0.40
		Task	10.97	5.48	*F*_2,139.61_ = 0.81	.4461	0.01
		Response × task	28.05	14.02	*F*_2,130.54_ = 2.08	.1295	0.03
	Theta power	Age	6.48	6.48	*F*_1,29.71_ = 4.47	.0429	0.13
		Accuracy	0.79	0.79	*F*_1,158.68_ = 0.54	.4624	0.00
		Response	165.98	165.98	*F*_1,129.36_ = 114.63	<.0001∗∗∗	0.47
		Task	5.81	2.90	*F*_2,135.45_ = 2.01	.1385	0.03
		Response × task	3.48	1.74	*F*_2,129.36_ = 1.20	.3046	0.02
	Theta ITPC	Age	0.02	0.02	*F*_1,29.17_ = 2.70	.1108	0.08
		Accuracy	0.15	0.15	*F*_1,74.33_ = 19.21	<.0001∗∗∗	0.21
		Response	0.03	0.03	*F*_1,130.97_ = 3.80	.053	0.03
		Task	0.10	0.05	*F*_2,144.32_ = 6.34	.0023∗	0.08
		Response × task	0.04	0.02	*F*_2,130.97_ = 2.76	.0668	0.04

∗*p* < .01, ∗∗*p* < .001, ∗∗∗*p* < .0001.

BD, bipolar spectrum disorder; EEG, electroencephalogram; ERP, event-related potential; HC, healthy control; ITPC, intertrial phase coherence; MS, mean of squares; SS, sum of squares; SZ, schizophrenia spectrum disorder.

### Group (HC, BD, SZ) Effects Within Each Task

**Figure 3:**
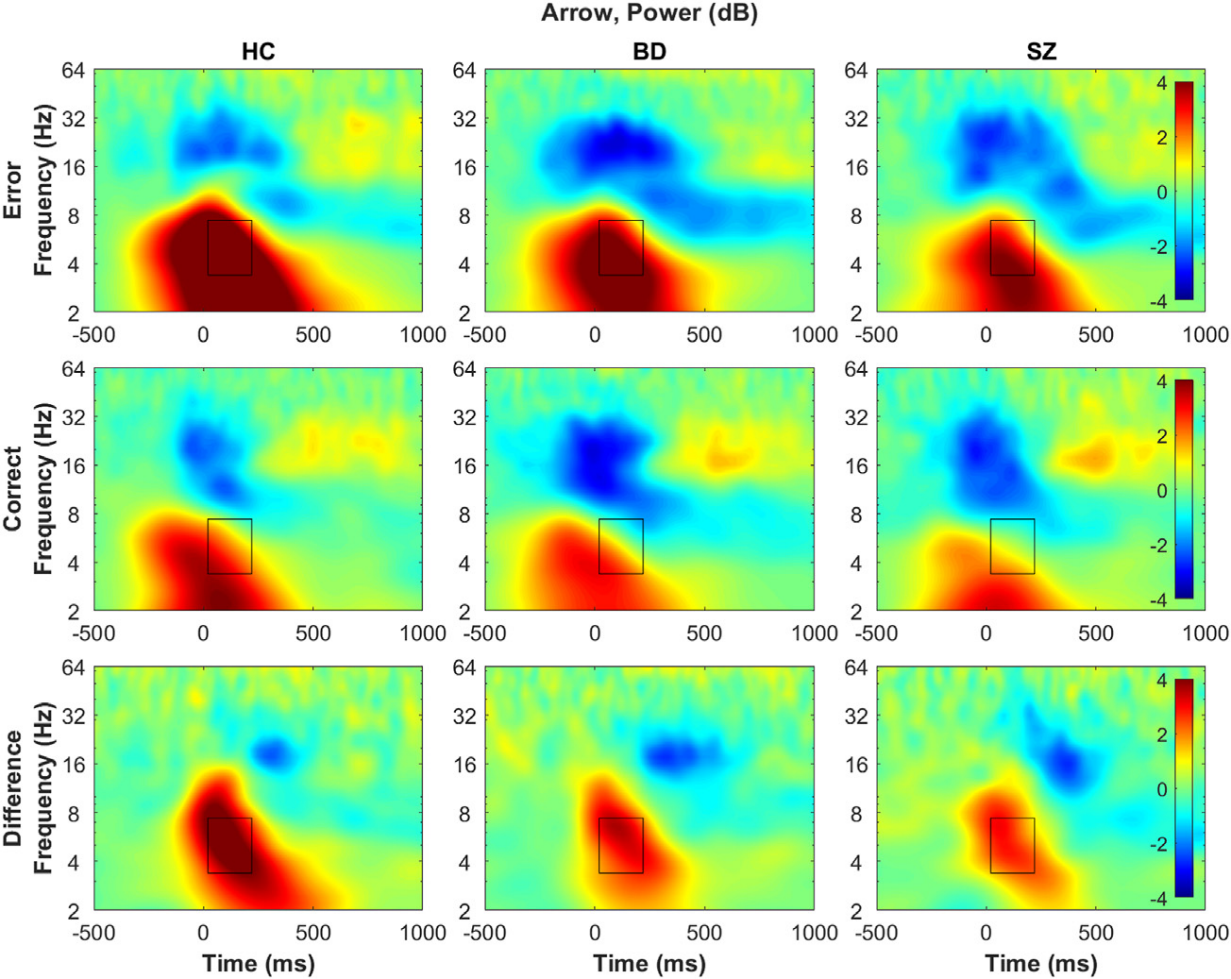
Time-frequency decomposition plots of total power at Cz electrode for the arrow task. Rows: top, error responses; middle, correct responses; bottom, difference between error and correct responses (error minus correct). Columns: left, healthy control (HC) group; middle, bipolar spectrum disorder (BD) group; right, schizophrenia spectrum disorder (SZ) group. The horizontal axis reflects time (−500 to 1000 ms relative to response; 0 ms indicates when response was made), and the vertical axis represents the frequency (2–64 Hz, logarithmically spaced). Heatmap is scaled from −4 dB (blue) to 4 dB (red) for all power plots for comparability. The boxed area represents the area that theta-band data was extracted from (20–220 ms and 3.38–7.38 Hz). Response main effects (larger theta power on error than correct responses) were found (Table 2). The SZ group showed reduced theta power in general (regardless of response type) compared with the HC group, and the BD group was not statistically significantly different from either group (Table S6). Line plots summarizing the findings are presented in Figure 2 (middle row). See the Supplement for time-frequency decomposition plots of total power from other tasks (Figures S4 and S5) and the scalp topography for power (Figure S9).

**Figure 4:**
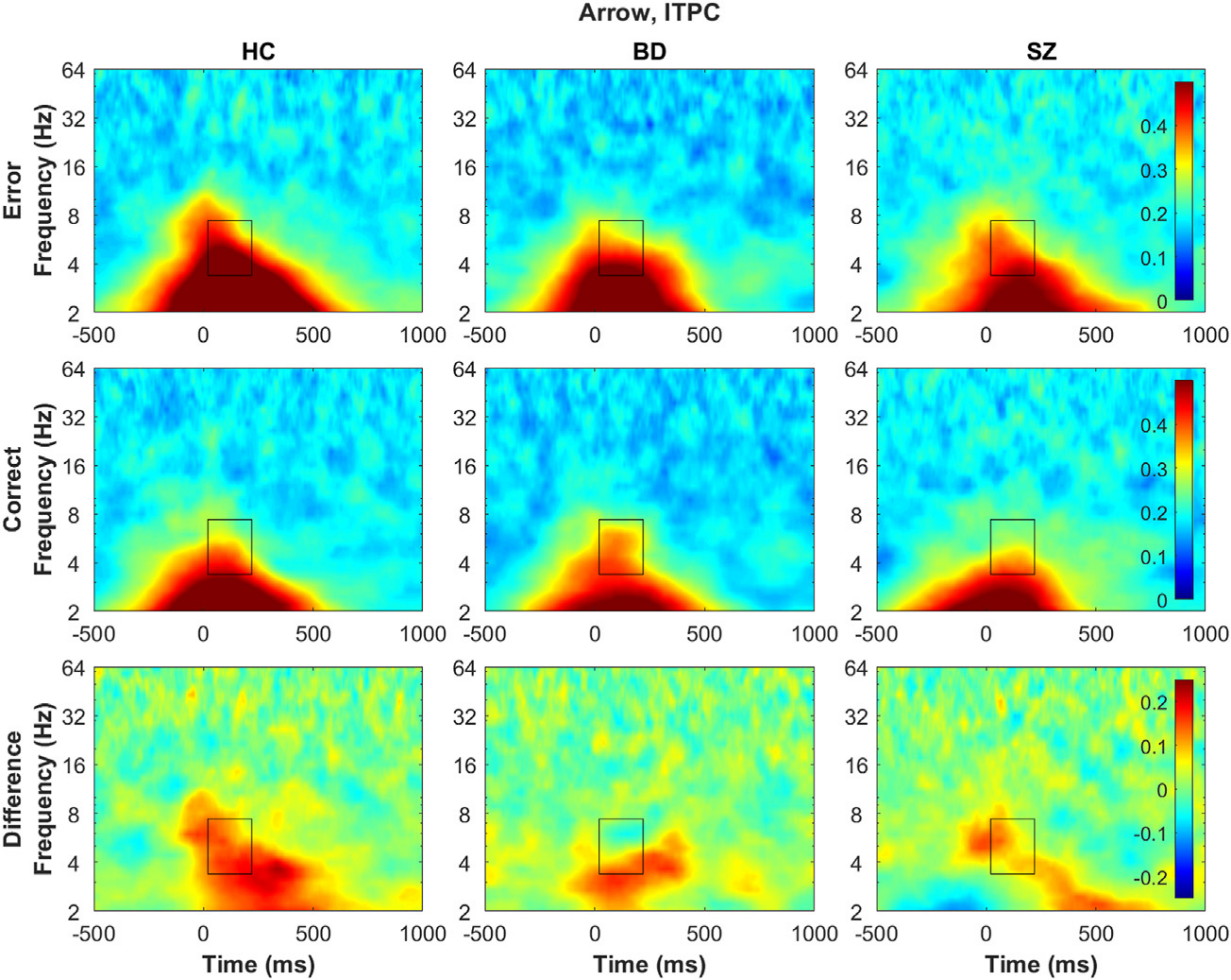
Time-frequency decomposition plots of intertrial phase coherence (ITPC) at Cz electrode for the arrow task. Rows: top, error responses; middle, correct responses; bottom, difference between error and correct responses (error minus correct). Columns: left, healthy control (HC) group; middle, bipolar spectrum disorder (BD) group; right, schizophrenia spectrum disorder (SZ) group. The horizontal axis reflects time (−500 to 1000 ms relative to response; 0 ms indicates when response was made), and the vertical axis represents the frequency (2–64 Hz, logarithmically spaced). Heatmap is scaled for 0 (blue) to 0.5 (red) for the error and correct responses and −0.25 to 0.25 for the difference. The boxed area represents the area that theta-band data was extracted from (20–220 ms and 3.38–7.38 Hz). There were no group differences in error theta ITPC (top row) or correct theta ITPC (middle row) (Table 2 and Table S6). In all groups, error theta ITPC was larger than correct theta ITPC (bottom row). Line plots summarizing the findings are presented in Figure 2 (bottom row). See the Supplement for time-frequency decomposition plots of ITPC from other tasks (Figures S6 and S7), and the scalp topography for ITPC (Figure S10).

In the 2-way ANCOVA for the arrow task, there was a main effect of group on theta power, such that theta power was smaller in the SZ group than in the HC group (*F*_2,76_ = 7.30, *p* = .0013, η^2^ = 0.16) (Figure 3), but the BD group did not differ from either group. For the unpleasant task, there was a group × response interaction effect (*F*_2,92_ = 6.36, *p* = .0026, η^2^ = 0.12) (Figure S4), such that error theta power was smaller in the SZ and BD groups than in the HC group, but correct theta power was comparable across groups. For the pleasant task, there was also a group × response interaction (*F*_2,80_ = 8.70, *p* = .0004, η^2^ = 0.18) (Figure S5), such that error theta power was smaller in the SZ group than in the HC group, but the BD group did not differ from either group, and correct theta power was comparable across groups. Furthermore, response effects were present in all analyses, except for ITPC in the unpleasant and pleasant tasks. There were no group effects on ERPs or theta ITPC in any task (all *F*s ≤ 0.39, *p*s ≥ .0525, η^2^ ≥ 0.07) (Figure 4 and Table 2; Figures S6 and S7; Table S6).

**Table 2:** Two-Way (Group × Response) Analyses of Covariance Results Within Each Task

Task	EEG Outcome	Predictor	SS	MS	*F*	*p*	η_p_^2^
Arrow	ERP	Age	43.05	43.05	*F*_1,76_ = 4.20	.0440	0.05
		Accuracy	0.02	0.02	*F*_1,76_ = 0.00	.9651	0.00
		Response	1461.55	1461.55	*F*_1,78_ = 142.46	<.0001∗∗∗	0.65
		Group	29.90	14.95	*F*_2,76_ = 1.46	.2394	0.04
		Response × group	26.34	13.17	*F*_2,78_ = 1.28	.2828	0.03
	Theta power	Age	2.08	2.08	*F*_1,76_ = 1.01	.3192	0.01
		Accuracy	2.08	2.08	*F*_1,76_ = 1.01	.3193	0.01
		Response	383.38	383.38	*F*_1,78_ = 184.90	<.0001∗∗∗	0.70
		Group	30.28	15.14	*F*_2,76_ = 7.30	.0013∗	0.16
		Response × group	17.88	8.94	*F*_2,78_ = 4.31	.0167	0.10
	Theta ITPC	Age	0.01	0.01	*F*_1,76_ = 0.79	.3761	0.01
		Accuracy	0.05	0.05	*F*_1,76_ = 4.85	.0307	0.06
		Response	0.27	0.27	*F*_1,78_ = 25.81	<.0001∗∗∗	0.25
		Group	0.06	0.03	*F*_2,76_ = 3.06	.0525	0.07
		Response × group	0.05	0.03	*F*_2,78_ = 2.44	.0938	0.06
Unpleasant	ERP	Age	39.31	39.31	*F*_1,90_ = 4.89	.0296	0.05
		Accuracy	4.06	4.06	*F*_1,90_ = 0.50	.4792	0.01
		Response	857.39	857.39	*F*_1,92_ = 106.64	<.0001∗∗∗	0.54
		Group	6.27	3.14	*F*_2,90_ = 0.39	.6781	0.01
		Response × group	35.92	17.96	*F*_1,92_ = 2.23	.1129	0.05
	Theta power	Age	3.86	3.86	*F*_1,90_ = 3.02	.0855	0.03
		Accuracy	1.54	1.54	*F*_1,90_ = 1.21	.2750	0.01
		Response	298.79	298.79	*F*_1,92_ = 234.26	<.0001∗∗∗	0.72
		Group	15.92	7.96	*F*_2,90_ = 6.24	.0029∗	0.12
		Response × group	16.22	8.11	*F*_2,92_ = 6.36	.0026∗	0.12
	Theta ITPC	Age	0.02	0.02	*F*_1,182_ = 2.12	.1475	0.01
		Accuracy	0.24	0.24	*F*_1,182_ = 24.16	<.0001∗∗∗	0.12
		Response	0.00	0.00	*F*_1,182_ = 0.02	.899	0.00
		Group	0.03	0.01	*F*_2,182_ = 1.39	.2519	0.02
		Response × group	0.00	0.00	*F*_2,182_ = 0.24	.7877	0.00
Pleasant	ERP	Age	20.53	20.53	*F*_1,78_ = 2.74	.1019	0.03
		Accuracy	0.07	0.07	*F*_1,78_ = 0.01	.9236	0.00
		Response	787.82	787.82	*F*_1,80_ = 105.13	<.0001∗∗∗	0.57
		Group	12.30	6.15	*F*_2,78_ = 0.82	.4440	0.02
		Response × group	25.73	12.87	*F*_2,80_ = 1.72	.1862	0.04
	Theta power	Age	12.59	12.59	*F*_1,78_ = 10.51	.0017∗	0.12
		Accuracy	7.36	7.36	*F*_1,78_ = 6.14	.0154	0.07
		Response	301.15	301.15	*F*_1,80_ = 251.42	<.0001∗∗∗	0.76
		Group	10.25	5.13	*F*_2,78_ = 4.28	.0172	0.10
		Response × group	20.83	10.42	*F*_2,80_ = 8.70	.0004∗∗	0.18
	Theta ITPC	Age	0.01	0.01	*F*_1,158_ = 0.69	.4075	0.00
		Accuracy	0.28	0.28	*F*_1,158_ = 30.91	<.0001∗∗∗	0.16
		Response	0.04	0.04	*F*_1,158_ = 4.49	.0356	0.03
		Group	0.03	0.01	*F*_2,158_ = 1.58	.2087	0.02
		Response × group	0.01	0.00	*F*_2,158_ = 0.54	.5849	0.01

There were singularity warnings for theta ITPC analyses using unpleasant and pleasant tasks. Follow-up analyses were conducted by reducing one variable at a time to identify the source of these singularities. For the unpleasant task, the removal of accuracy and response × group interaction alleviated this issue. For the pleasant task, the removal of accuracy alleviated this issue. The results for the remaining variables did not change.

∗*p* < .01, ∗∗*p* < .001, ∗∗∗*p* < .0001.

EEG, electroencephalogram; ERP, event-related potential; ITPC, intertrial phase coherence; MS, mean of squares; SS, sum of squares.

### Group × Task Interaction Effects

In the 3-way ANCOVA (Table S7), there were no group × task or response × group × task interaction effects, supporting the interpretability of the 2-way ANCOVAs.

### Covariates and Correlations

**Figure 5:**
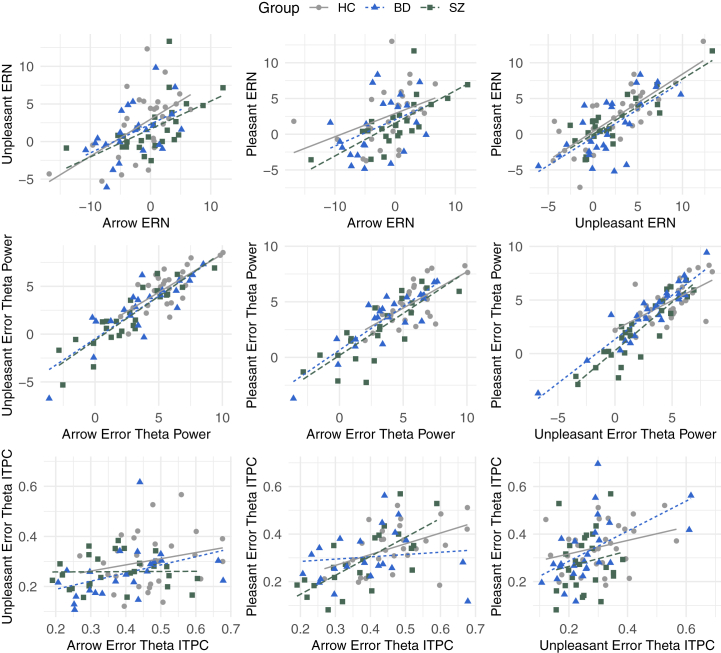
Selected scatterplots showing associations between error-related electroencephalogram measures elicited by different tasks (see Table S8 for all correlation coefficients), with estimated regression lines in each group. Rows: top, error-related negativity (ERN); middle, error-related theta power; bottom, error-related intertrial phase coherence (ITPC). Columns: left, scatterplot between arrow and unpleasant tasks; middle, scatterplot between arrow and pleasant tasks; right, scatterplot between unpleasant and pleasant tasks. BD, bipolar spectrum disorder; HC, healthy control; SZ, schizophrenia spectrum disorder.

Age was not related to any EEG indicators, except theta power in the pleasant task analysis. Accuracy was related to ITPC in all analyses, except the arrow task analysis. Accuracy was not related to any other EEG metrics. Correlations among EEG indicators (Figure 5 and Table S8) suggest that ERPs across responses and tasks tended to correlate with each other at a comparable rate as previously reported (median *r*s are reported; same task = 0.60; different tasks = 0.55) (41,57). However, theta power showed slightly stronger correlations (same task = 0.73; different tasks = 0.74), while theta ITPC showed much weaker correlations (same task = 0.09; different tasks = 0.16). Intraclass correlation coefficients (ICCs) suggest very little to no task effect on power but consistent task effects on ITPC, especially on error trials (Table S9). There were no consistent correlational patterns of EEG indicators with behavioral data and clinical scales (Table S10).

## Discussion

In this study, we investigated cognitive control, operationalized as response-monitoring ERPs, theta-band power, and theta-band ITPC, among individuals with SZ and BD using flanker tasks. Furthermore, the EEG indicators were elicited using the traditional arrow (i.e., emotionally neutral) and emotional (i.e., unpleasant, pleasant) stimuli to investigate the role of emotion appraisal on response monitoring. The findings generally converge with previous literature: 1) emotion appraisal modulates EEG activities (although in the opposite direction as hypothesized), and 2) the general pattern suggests that individuals with SZ show reduced EEG activities compared with HC participants, and individuals with BD were in between. Furthermore, theta power and ITPC presented a nuanced picture that was not apparent in ERPs and potential differential cognitive functions in emotional contexts.

### Between-Group ERP

ERPs (ERN, CRN) were modulated by emotional stimuli in HC participants and individuals with BD, but not in individuals with SZ. This lack of modulation by task is consistent with previous research (38) and may reflect the difficulty that individuals with SZ have engaging cognitive control across different contexts. Contrary to our hypothesis and previous finding with college students (41), emotion appraisal attenuated ERPs in HC participants, which may reflect the difficulty engaging cognitive control in older individuals (see the Supplement for more discussion). Furthermore, neither the ERN nor CRN was reduced in individuals with SZ, which was also contrary to previous findings (25,38) and our hypothesis. This may reflect the relatively small sample size or task design differences from previous studies (25,38). For example, higher accuracy is associated with a larger ERN (58), and presentation time was adjusted in the current study to maintain accuracy comparable across participants. More importantly, given the theta power and ITPC findings, the lack of consistency may reflect limitations of ERPs as they reflect the sum of all time-frequency activities.

### Between-Group Theta

Theta power, particularly on error trials, was reduced in individuals with SZ across all tasks and in individuals with BD in the unpleasant task. Because theta ITPC was not reduced in either clinical group, theta power may be a specific EEG indicator of the broad cognitive deficits in SZ and BD [e.g., (14)] and less susceptible to the impact of emotion appraisal. Such findings of theta power’s stronger relevance to psychopathology than ITPC has been reported with OCD (43). Thus, theta power may reflect the effort or ability of cognitive engagement most affected by psychopathology, such as the hypothesized role of the ERN reflecting an alarm system (22). When interactions involving response type (error, correct) emerged, only the lack of increase in EEG activities on error trials seemed to underlie the deficits in clinical groups. These findings could indicate that some routine cognitive control may be intact (when no correction is needed), but the ability to engage cognitive control when needed is impaired (correcting for mistakes) (61). While participants with BD showed similar patterns to HC participants in ERPs and in theta power on the arrow and pleasant tasks, they had a similar pattern to participants with SZ for theta power on the unpleasant task and ITPC. These add to previous research findings that individuals with BD have some cognitive control impairments, although to a lesser degree than individuals with SZ (8,15). The diagnosis-agnostic cluster analysis based on EEG theta measures also suggested that the boundaries between SZ and BD are not clear, and history of psychosis symptoms (less prevalent in BD) may more strongly be related to the underlying endophenotype [e.g., (62)] (see the Supplement for more discussion).

While theta power was not modulated by emotional stimuli in any group, ITPC was for all groups, suggesting that ITPC underlies the ERP modulation by stimuli. ITPC may reflect a mechanism that is intact in SZ and BD, such as the intact appraisal of emotional images despite the lack of expression (39,63,64). While power may reflect an alarm system, phase activities have been shown to be related to interregional communication (48,49) and processing efficiency from computational modeling (65) and thus may be involved in utilizing brain dynamics. For example, the accuracy covariate was consistently related to ITPC, reflecting the importance of phase activities in regulating downstream behaviors. Furthermore, ITPC did not differ between error and correct trials in the BD and SZ groups and also in the unpleasant and pleasant tasks (including HC group). These may collectively suggest that, in individuals with BD and SZ, the cognitive control mechanism highly influenced by emotion appraisal is constantly in a state similar to that of healthy individuals dealing with emotional information, potentially reflecting stress sensitivity (32). Phase activity has not been as commonly investigated as power (45), and further research is needed to contextualize the role of phase in psychopathology.

### Within-Group Individual Differences

The correlations among EEG indicators provide another perspective and a psychometric strength of theta activity as an individual difference measure. The ERN and CRN correlations were similar to those that have been reported using multiple tasks in nonclinical samples (approximately 0.40–0.60) (41,57), indicating the existence of a general tendency to elicit the ERN across tasks but also task-specific variances (66). Theta power had stronger correlations across tasks (median approximately 0.70), while ITPC did not (median approximately 0.10). ICCs also suggest similar task and individual effects, especially on error trials. Thus, theta power may reflect general cognitive control while phase may reflect task-specific processes. Theta power has been proposed to integrate the literature on ERPs elicited from various tasks (45,67). These findings suggest that using power may be appropriate in integrating findings across tasks and research domains (e.g., animal models). More psychometric research on theta (and other time-frequency) activities are needed for thorough integration of the ERP literature. EEG measures did not consistently relate to clinical or behavioral measures, although theta power related to RT variables across tasks in SZ (Table S10). These likely reflect the complex neurological mechanisms that underlie observed behaviors and symptoms, such that midfrontal theta activities alone are not sufficient to understand such relationships. However, interregional and cross-frequency relationships show promise to reflect complex neurological dynamics (48,49) that may elucidate the brain dynamics that underlie behaviors and clinical symptoms.

### Limitations

The sample sizes were relatively small (approximately 30 per group), especially for correlational analyses that were conducted separately for each group, and some null findings may reflect type II errors. While a conservative statistical significance threshold (*p* < .01) was used to interpret results, this does not control for the entirety of the multiple analyses conducted. The clinical groups were relatively heterogeneous, and there might have been some overlap (e.g., schizoaffective vs. bipolar I disorder). Psychotropic medication was taken by most participants with BD and SZ, although there were no antipsychotic effects on EEG measures in this small sample (Table S11). Limited literature investigating the effects of atypical antipsychotics and lithium indicates that they may enhance ERN and theta power (68,69); thus, the reduction in this sample likely does not reflect medication effects. While age was included as a covariate in all analyses and did not relate to any EEG measures in this study, the range was wide (18 to 65). Task order is another potential confound because participants might have been more fatigued during the emotional tasks that were administered later than the arrow task. However, a previous study that used a similar paradigm found that, regardless of the order, emotional stimuli uniquely affected cognitive control compared with face stimuli, likely indicating specific emotion appraisal effects (41). Some self-reported task affect (Tables S12 and S13), particularly for the pleasant task, suggests that the emotional stimuli did not have as strong an intended effect and may underlie some of the null task effects.

### Conclusions

This project collected novel response-monitoring ERPs (ERN, CRN), theta power, and theta phase data among individuals with SZ and BD in 3 variants of flanker tasks that ranged in emotional valence. Individuals with SZ did not show the same modulation by emotion on ERPs that were present in the HC and BD groups. However, nuances emerged when we investigated theta-band activities, such that theta power was blunted in individuals with SZ while ITPC was modulated by task in all 3 groups. Individuals with BD showed patterns sometimes similar to individuals with SZ and sometimes similar to HC participants. This study highlights the benefit and importance of the transdiagnostic investigation of multiple diagnostic groups and multiple tasks concurrently.

## Supplementary Material

Supplemental Text, Figures S1–S12, and Tables S1–S18

Key Resource Table
