# Supplementary material for: Response Monitoring Theta-Band Activities Across Emotional Contexts in Schizophrenia and Bipolar Spectrum Disorders
Source: Biol Psychiatry Glob Open Sci. 2025 May 27;5(5):100540. doi: 10.1016/j.bpsgos.2025.100540 (PMC12272877; doi:10.1016/j.bpsgos.2025.100540)
Supplement: Supplemental Text, Figures S1–S12, and Tables S1–S18 [file mmc1.pdf]

SUPPLEMENTARY INFORMATION

Response Monitoring Theta-Band Activities Across Emotional Contexts in Schizophrenia and Bipolar Spectrum Disorders

Suzuki *et al.*

Supplementary Materials

Additional Methods Information

Participants. The Bipolar Disorder (BD) group was defined as participants with the diagnosis of bipolar disorder I, II, or other specified/unspecified. The Schizophrenia (SZ) group was defined as participants with the diagnosis of schizophrenia, schizoaffective disorder, or other specified/unspecified psychosis.

Participants aged 18—65 were recruited from clinical sites and research registries at the Michigan Medicine Department of Psychiatry, referrals from other researchers, the University of Michigan Health Research website (UMHealthResearch.org), and community advertisements. A total of 34 healthy control (HC), 33 BD, and 32 SZ participants enrolled in the study. The final sample consisted of 33 HC, 33 BD, and 32 SZ participants with usable EEG data from at least one of the three flanker tasks. The specific numbers of participants per group with usable EEG data for each of the three flanker tasks is reported in Tables S3 and S4.

Exclusion criteria for all participants included history of serious neurological illness or current medical condition that could compromise brain function (e.g., liver failure), history of closed head injury (i.e., loss of consciousness > 5 minutes, hospitalization, or neurological sequelae), diagnosed intellectual disability, meeting criteria for substance use disorder within the past month at the time of diagnostic assessment and current active suicidal thoughts with plans or intentions, as assessed by Columbia Suicide Severity Rating Scale (1). All participants had normal or corrected-to-normal eyesight (at least 20/30) according to a Snellen Test and had a smartphone, computer, or other way to access the internet to complete survey study measures remotely. Additional exclusion criteria for HC included personal lifetime history of a DSM-5 axis-I disorder, except for simple phobias and substance use disorders that remitted at least 1 year prior to assessment. The Structured Clinical Interview for DSM-5—Research Version (SCID-5; 2) was used to assess participant diagnosis. If it was available to research staff, clinical information from the electronic health record (e.g., diagnostic impressions made by treating clinicians) and/or interview data from prior study participation were used to supplement the interviews and determine diagnoses (e.g., Prechter Longitudinal Study of Bipolar Disorder; 3,4). All clinical interviews were administered via Zoom video teleconferencing in advance of participants' lab visit, to minimize in-person lab time and reduce risk of Covid-19. All interviews were conducted by MWM (master's level clinical psychology doctoral candidate) and reviewed with TS (doctoral level psychologist). All data was collected between October 2021 and June 2023.

While there were on inclusion or exclusion criteria based on mood at time of study participation, the mood states of participants are reported in Table S14. For this summary, we use the following threshold: HAMD-17 < 8 and YMRS < 8 to indicate euthymia; HAMD-17 $\geq$ 8 as depressed; YMRS $\geq$ 8 as manic or hypomanic; and both HAMD-17 and YMRS $\geq$ 8 as mixed (5,6).

Task Programming Error
If participants performed at 90% or better in the practice and first nine blocks, the stimulus presentation time for the 10th block became “0ms”. However, the program interpreted such presentation time to keep the stimuli on screen until participants responded. This affected 2 participants in the arrow task, and 1 in the Unpleasant and Pleasant flanker tasks. Given the small proportion of the data and participants affected (10% of the data in 3 participants), analyses proceeded without removal of these participants or their 10th blocks.

Task Administration
Arrow flanker task was always administered first because this task serves as the common task with other ERN and response-monitoring related research and has the simplest instruction to ensure that the participants could complete a speeded-response flanker decision making task. Order of Unpleasant and Pleasant tasks were counterbalanced to ensure there was no ordering effect between these tasks.

Task Effect Validation
Participants appraised the unpleasantness, pleasantness, and excitability of the tasks using a modified visual analog scale (7). Specifically, immediately after the completion of each task, participants were instructed to rate the unpleasantness, pleasantness, and excitability of each task (“During the task, I felt…”) on a 0 (“not at all unpleasant”, “not at all pleasant”, and “calm”, respectively) to 100 (“very unpleasant”, “very pleasant”, and “excited”, respectively) scale. Descriptive statistics of these ratings in each diagnostic group are in Table S12.

Nine one-way ANOVAs were conducted to compare the three ratings (feeling pleasant, feeling unpleasant, feeling excited) across all three tasks (arrow, unpleasant, pleasant), separately within each diagnostic group (Table S13). Using .01 as a significance threshold, the HC and BD groups rated the unpleasant task as more unpleasant than the arrow or pleasant task, and less pleasant than the pleasant task. The BD group also rated the unpleasant task as less pleasant than the arrow task. In the SZ group, the only difference reaching significance was that the unpleasant task was more unpleasant than the pleasant task. All tasks had comparable excitement ratings in all groups.

EEG Data Acquisition
EEG data was collected using a 64-electrode BrainVision actiCap and actiChamp system (Brain Products, GmbH) while participants completed the tasks. Data was collected at 2500Hz with a Cz online reference. Impedance was kept at or below 25 kΩ.

EEG Data Preprocessing
Custom scripts using MATLAB (8) and EEGLAB (9) for automatic artifact rejection algorithm based on Delorme (10) was used. Specifically, data was down sampled to 500 Hz, periods without any event for more than 5 seconds (e.g., between blocks) were removed, and only the first response after a trial were retained (e.g., the second response by participants trying to correct the error response were removed). Data to calculate behavioral variables (e.g., accuracy) were extracted at this point. Additional responses that were too fast (100ms or quicker; or 3 median absolute deviation [MAD] below median reaction time [RT]) and too slow (1500ms or slower; or 3 MAD above median RT) were removed. EEG data was re-referenced to the average

of all electrodes, bandpass filtered (0.1 Hz to 249 Hz; 11), and 60 Hz line noise and harmonics were removed using the cleanline package (12). “Bad” channels and data segments were removed using the pop_clean_rawdata function of EEGLAB. Bad channels, defined as less than .80 correlation with nearby electrodes, were removed and data was average referenced again. Bad data segments, defined as artifact subspace reconstruction (ARS) parameter of 100 or above, were removed. ICA was conducted on this data and IClabel (13) was used to identify and remove components with 75% or higher chance of being ocular artifacts. Data were epoched, baseline corrected to -200 to -50ms relative to response, and segments containing voltage > ±200 µV in any channel between 0 to 500ms post-response were removed. Parameters were selected to optimize data cleanliness and retention of trials in this dataset prior to any EEG extraction.

Below are the descriptive statistics of the number of channels and independent components removed through the automatic preprocessing pipeline.

	Number of channels removed/interpolated	Number of components removed
Arrow	M = 6.43 (SD = 5.09)	M = 5.53 (SD = 4.10)
Unpleasant	M = 5.13 (SD = 3.88)	M = 6.08 (SD = 4.37)
Pleasant	M = 5.10 (SD = 3.76)	M = 6.18 (SD = 4.42)

For each task, only participants with above 65% accuracy and at least eight error trials remaining at the end of all preprocessing steps were included in subsequent analyses. The eight-trial threshold was chosen to balance sufficient reliability/signal-to-noise ratio (improved with higher threshold) while including as many participants as possible (improved with lower threshold), the latter of which is more important in detecting task- and group-effects (14–16).

R packages used

The following R packages were used: plyr (v1.8.9; 17) for behavioral data calculations, dplyr (v1.1.4; 18) and reshape2 (v1.4.4; 19) for data management, psych (v2.4.6.26; 20) for descriptive statistics and correlations, lme4 (v1.1-35.5; 21) and lmerTest (v3.1-3; 22) for ANCOVAs, emmeans (v1.10.4; 23) for post-hoc contrasts, effectsize (v. 0.8.9; 24), base stats for other analyses, ggplot2 (v3.5.1; 25) for plotting.

Changes from pre-registration

Some aspects of the methodology were adjusted from the pre-registration (https://osf.io/75nhw), which are described below. Generally, we aimed to minimize data preprocessing and expand on variables that we could analyze for consistency in data used with future manuscripts.

1. Bandpass filter range change from the proposed 0.1 –100 Hz to 0.1 –249 Hz to minimize altering the data from preprocessing.
2. Complex Morlet wavelet convolutions range changed from the proposed 2 to 80 Hz, to 2 to 128 Hz to include higher gamma range data.
3. Time-frequency decomposition was applied to all channels instead of the only three proposed.
4. Proposed statistical analyses focused only on the error trials, and no response effect was proposed to be analyzed. We changed this to include both error and correct trials to test for response main and interaction effects, and to add statistical power.

5. The 3-way omnibus ANCOVA analysis was not proposed but was conducted for comprehensiveness.

Results from Correlations of EEG Indicators with Behavioral Data and Clinical Scales
Correlations between EEG measures with behavioral and clinical measures are presented in Table S10. As stated in the main text, there was no simple pattern, but individuals with SZ showed stronger relations between theta power and behavioral measures than the other two groups, suggesting potential higher relevance of individual differences of EEG power with SZ than BD or HC (general population).

Discussion on comparison to previous study using non-clinical undergraduate sample
While both emotional images modulated the EEG measures, the direction was contrary to our hypothesis and previous findings that emotional images elicited larger activity than neutral (26). One potential source of difference is the pre-*response* baseline used for calculating the ERP measures. In this study, a baseline more proximal to the response (-200 to -50ms) than the previous study (-400 to -200ms). When an earlier baseline was used on this data, Pleasant task showed larger ERN and Unpleasant was comparable to Arrow task (In HC-only analysis, ERNs were comparable across all tasks; Table S15), suggesting that the earlier baseline "increased' the ERPs of emotional tasks relative to the Arrow task. The more proximal ERP baseline findings were more aligned to theta activities, and was chosen for interpretation in this study (27).The baseline selection, however, does not fully resolve the direction of emotional modulation contrary to our expectations in all EEG indicators.

Age of participants were another key difference between the previous (Mean Age = 19.18; 26) and this study (HC Mean Age = 36.91) investigating the emotional effects on the ERN and may provide insight into what the theta activities reflect. EEG activities generally increase with age until early adulthood then decrease with age (28,29, but see 30). In this task, the emotional images may have been more distractive than the arrow stimuli, such that older participants had difficulty re-allocating cognitive resources to response-monitoring (31,32) while undergraduate student participants were able to, potentially to the point of overactivation. Similarly, older participants in this study may have been able to compensate for relatively simple stimuli (i.e., arrow), while it was difficult for them to compensate for any emotional stimuli.

Discussion on cluster analysis agnostic to diagnostic status
A cluster analysis based on the theta EEG measures were conducted using 72 participants with all EEG measures. Principal component analysis was conducted and parallel analysis suggested that at least 2 components be extracted. Component 1 was characterized by high loadings from most EEG measures, except for theta ITPC correct trials (Table S16). Component 2 is characterized by high loadings from theta ITPC correct trials. Component 3 is primarily characterized by high loadings from theta ITPC error trails and arrow theta power correct trials, and were less interpretable than the first two components that appear to reflect reflecting reciprocal measures.

K-means cluster analysis was conducted on this group to identify diagnostically agnostic clusters/groups. Two- and three-cluster memberships were extracted and crosstab tables of memberships across the two solutions and diagnostic groups are summarized in Table S16.2. Results suggest that, in the 2-cluster solution, most HC participants were in Group 2, while the clinical groups were split into both groups. The 3-group solution indicate that Group 1 was

characterized by a mix of the three diagnostic groups, Group 2 was characterized primarily by HC participants, and Group 3 was primarily characterized by SZ participants. Scatterplots (Figure S11) represent the distribution of the 3-group solution and diagnostic group distribution on the two components.

T-tests and ANOVAs to test the group differences on the clinical measures and chi-square tests to test frequency differences in those with psychosis were conducted (Table S16.3). The two-cluster solution indicated that the cluster characterized by clinical participants were higher on HAMD and PANSS General scores and mostly (80%) with psychosis symptoms. The three-cluster solution indicated that the cluster characterized primarily by SZ participants were higher on PANSS General score than the other two clusters, and there were no group differences on other measures. The results suggest that the theta-band measures (except ITPC correct trials) may reflect a potential endophenotype, although further research is needed.

ERP calculations using all trials and the results

Results using all trials (non-matched trials) are presented in Tables S17 and Figure S12. This represents the traditional calculation of the ERN and CRN. The overall results did not appreciably differ from matched trials (Tables 1, 2, S5, and S6), although the planned contrast of groups in the Arrow task suggest participants with SZ had generally lower ERP amplitude than those with BD (Same direction but not statistically significant in Table S6).

Root mean square of the standardized measurement error: RMS(SME)

To quantify data quality of the ERPs and theta-band power, standardized measurement error (SME) was calculated for each participant (33). The SME is the calculated as standard deviation of the EEG metric (SD) across tasks, divided by the square root of number of trials. SME can be interpreted as standard error of measurement, which makes it an intuitive measure to interpret. The SME calculated for all participants were then aggregated by taking the root mean square (RMS) of the SME to calculate the RMS(SME). This provides a singular value to quantify the variance of the EEG data. The RMS(SME) values for all ERPs and theta-power for each task are reported in Table S18. Further, signal-to-noise ratio (SNR), quantified as the mean amplitude or value of the EEG metric divided by RMS(SME) are also reported in Table S18. Negative SNR values suggest that the mean amplitude was a negative value.

Table S1. Catalog numbers of the International Affective Picture System images used in the emotional flanker tasks.

Task	Image Type	Used Blocks	Catalog Numbers
Unpleasant	Unpleasant	Practice	1525, 3030, 6230, 6313
		Main	1120, 1301, 1304, 1930, 3001, 3015, 3016, 3019, 3051, 3061, 3062, 3140, 3150, 3168, 3185, 3195, 3261, 6231, 6242, 6244, 6315, 6370, 6560, 6561,9425
	Neutral	Practice	5726, 7165, 7489, 7545
		Main	5390, 5510, 5520, 5740, 7000, 7006, 7010, 7041, 7050, 7055, 7062, 7090, 7096, 7100, 7150, 7170, 7185, 7490, 7495, 7504, 7546, 7590, 7595, 7710, 9360
Pleasant	Pleasant	Practice	1463, 2040, 4652, 4800
		Main	1710, 1722, 2045, 2058, 2071, 2150, 2160, 2208, 2209, 2303, 2345, 2347, 4608, 4656, 4658, 4659, 4660, 4664, 4687, 4689, 4693, 4694, 4697, 4698, 4810
	Neutral	Practice	5750, 7078, 7136, 7510
		Main	5395, 5471, 5530, 5531, 5731, 7002, 7004, 7025, 7034, 7035, 7040, 7053, 7056, 7061, 7077, 7081, 7095, 7161, 7175, 7491, 7500, 7547, 7560, 7700, 9468

Table S2. Descriptive statistics of participant demographic and clinical measure variables for each group.

	**HC** *N* = 33				**BD** *N* = 33				**SZ** *N* = 32							
	M	*SD*	*Min*	*Max*	*M*	*SD*	*Min*	*Max*	*M*	*SD*	*Min*	*Max*	F or $\chi2$	*df*	*p*	*pairwise contrasts*
**Demographic**																
**Age**	36.91	15.00	18.00	63.00	38.67	14.88	18.00	65.00	40.22	13.74	18.00	64.00	0.42	2, 95	.6579	
**Sex** (M / F)	13 / 20				7 / 26				12 / 20				2.99	2	.2245	
**Gender** (M / F / Other)	13 / 20 / 0				8 / 21 / 4				15 / 13 / 4				8.26	4	.0825	
**Race** (White / Black or AA / Asian / Multiracial / Other)	25 / 2 / 4 / 1 / 1				27 / 3 / 3 / 0 / 0				24 / 5 / 1 / 1 / 1				0.53	2	.7688	
**Ethnicity** (Hispanic or Latino / Non Hispanic or Latino)	1 / 32				4 / 29				1 / 31				3.12	2	.2106	
**Education** (yrs)	16.79	2.19	13.00	21.00	16.00	1.64	12.00	19.00	15.64	2.14	12.00	21.00	2.79	2, 95	.0663	
**Parental education** (yrs)	15.49	2.76	10.00	20.00	14.23	2.74	7.00	18.50	14.91	2.49	12.00	19.50	1.86	2, 95	.1612	
**Clinical**																
**Diagnosis**	--				21 BDI / 7 BDII / 5 BD-Other				11 Schizophrenia / 16 SZA / 5 SZ-other					--		
**HAMD-17** (0–52)	0.64	0.93	0.00	3.00	7.33	6.48	0.00	20.00	6.50	5.74	0.00	20.00	17.38	2, 95	<.0001	HC < BD, SZ
**YMRS** (0–60)	0.21	0.48	0.00	2.00	4.27	4.38	0.00	16.00	5.25	4.38	0.00	17.00	18.21	2, 95	<.0001	HC < BD, SZ
**PANSS-Positive** (7–49)	7.06	0.24	7.00	8.00	9.33	2.31	7.00	16.00	12.78	4.05	7.00	23.00	37.44	2, 95	<.0001	HC < BD < SZ
**PANSS-Negative** (7–49)	7.50	1.97	7.00	18.00	8.97	3.31	7.00	21.00	12.06	5.33	7.00	24.00	12.09	2, 94	<.0001	HC, BD < SZ
**PANSS-General** (16–112)	16.15	0.51	16.00	18.00	22.64	6.24	16.00	34.00	24.72	5.69	16.00	40.00	27.45	2, 95	<.0001	HC < BD, SZ
**Medication** (Taking / Not taking)																
Antipsychotic					20 / 13				27 / 5				3.47	1	.0624	
Lithium					4 / 29				5 / 27				0.002	1	.9603	
Anticonvulsant					19 / 14				10 / 22				3.55	1	.0594	
SSRI/SNRI					11 / 22				12 / 20				0.01	1	.9269	
Stimulant					5 / 28				1 / 31				1.55	1	.2127	
Benzodiazepine					2 / 31				6 / 26				1.391	1	.2383	

Notes. For group labels, HC = Healthy control group; BD = Bipolar spectrum disorder group; SZ = Schizophrenia spectrum disorder group (SZ); for specific diagnoses, BD = Bipolar disorder, SZ = Schizophrenia, SZA = Schizoaffective disorder, BD-other and SZ-other refer to other specified/unspecified bipolar disorder and psychosis, respectively; M = Mean; SD = Standard Deviation; Min = Minimum Value; Max = Maximum value; *df* = Degrees of freedom (numerator, denominator); *p* = Statistical significance; HAMD = Hamilton Depression Rating Scale; YMRS = Young Mania Rating Scale; PANSS = Positive and Negative Syndrome Scale; SSRI = Selective serotonin reuptake inhibitors; SNRI = Serotonin and

norepinephrine reuptake inhibitors. Sample reported on above is all participants with at least usable EEG data for at least one task. Race chi-square test is based on white vs non-white due to small cell counts in others. For all clinical scales, possible range of scores is in parentheses after name of scale.

Table S3. Descriptive statistics of EEG measures for each group.

			HC				BD				SZ			
	N for Arrow / Unpleasant / Pleasant task		29 / 33 / 30				25 / 31 / 27				27 / 31 / 26			
	**Task**	**EEG Measure**	*M*	*SD*	*Min*	*Max*	*M*	*SD*	*Min*	*Max*	*M*	*SD*	*Min*	*Max*
**ERP amplitudes (µV)**	**Arrow**	ERN	-1.21	4.81	-16.83	6.66	-2.63	4.33	-10.76	5.14	0.28	5.14	-13.97	12.04
		CRN	5.18	4.97	-5.15	13.42	4.14	4.83	-2.63	19.44	5.17	4.52	-5.39	14.49
		CRN all trials	3.62	3.64	-2.55	9.81	1.42	3.16	-3.82	8.16	3.67	3.84	-3.28	12.24
		ΔERN	-6.39	5.09	-16.93	2.84	-6.78	4.12	-17.46	-1.34	-4.89	4.24	-13.32	3.07
		ΔERN all trials	-4.88	4.58	-15.33	2.86	-4.06	3.41	-9.35	6.14	-3.39	4.49	-12.84	2.56
	**Unpleasant**	ERN	2.07	4.23	-5.27	12.33	1.49	3.51	-6.09	9.82	1.84	3.52	-4.19	13.33
		CRN	7.00	3.96	0.01	16.59	6.29	5.73	-0.65	23.57	4.86	4.35	-7.34	18.07
		CRN all trials	6.16	3.59	-0.57	16.84	5.06	4.11	-0.07	17.65	4.18	3.43	-2.15	15.84
		ΔERN	-4.93	3.37	-12.19	2.40	-4.81	5.21	-21.19	3.98	-3.02	3.18	-9.64	9.59
		ΔERN all trials	-4.17	3.26	-13.00	0.51	-3.62	3.33	-15.16	0.88	-2.37	2.21	-6.67	4.39
	**Pleasant**	ERN	2.30	4.22	-7.43	13.00	0.78	4.25	-5.20	8.34	1.64	3.34	-3.57	11.67
		CRN	6.75	4.55	-2.91	19.72	6.09	4.92	-2.92	16.91	4.98	3.79	-1.34	14.29
		CRN all trials	6.39	3.94	0.22	18.87	5.36	4.15	-1.86	16.28	4.22	3.63	-2.96	14.19
		ΔERN	-4.45	4.10	-12.73	7.20	-5.31	4.46	-22.12	0.38	-3.34	2.79	-8.47	2.59
		ΔERN all trials	-4.17	3.61	-14.00	2.34	-4.62	4.30	-21.01	1.63	-2.59	2.68	-7.82	2.02
**Theta Power (dB)**	**Arrow**	Error	5.67	1.86	2.01	10.05	3.67	2.90	-3.61	8.51	2.80	2.96	-2.84	9.39
		Correct	1.71	1.71	-0.49	8.61	0.78	2.39	-7.17	4.49	0.40	1.87	-3.07	4.04
		ΔPower	3.96	2.02	-1.07	9.34	2.89	1.99	-0.78	6.04	2.40	2.10	-2.25	7.41
	**Unpleasant**	Error	4.84	2.08	0.01	8.52	2.85	3.15	-6.73	8.04	1.80	3.07	-5.31	6.91
		Correct	1.56	1.81	-2.23	8.09	0.48	2.33	-7.21	5.48	-0.08	2.40	-7.21	4.13
		ΔPower	3.28	1.67	-0.12	7.37	2.37	1.80	-0.64	5.67	1.88	1.27	0.17	4.73
	**Pleasant**	Error	4.83	1.82	1.52	8.24	3.79	2.73	-3.70	9.43	2.00	2.88	-2.87	6.84
		Correct	1.47	1.30	-1.86	4.09	0.76	2.20	-6.85	4.30	0.29	1.90	-2.81	3.75
		ΔPower	3.36	1.45	0.91	7.02	3.04	1.68	0.45	7.58	1.70	1.52	-0.65	4.37
**Theta ITPC**	**Arrow**	Error	0.47	0.10	0.27	0.68	0.39	0.13	0.21	0.68	0.38	0.12	0.19	0.60
		Correct	0.34	0.11	0.19	0.57	0.34	0.10	0.18	0.52	0.31	0.11	0.16	0.67
		ΔITPC	0.13	0.14	-0.17	0.44	0.05	0.17	-0.25	0.48	0.07	0.13	-0.11	0.43
	**Unpleasant**	Error	0.32	0.12	0.12	0.69	0.27	0.12	0.11	0.62	0.26	0.07	0.16	0.42
		Correct	0.30	0.10	0.13	0.51	0.28	0.12	0.13	0.56	0.26	0.10	0.13	0.51
		ΔITPC	0.01	0.18	-0.39	0.42	-0.01	0.14	-0.30	0.32	-0.01	0.12	-0.25	0.22
	**Pleasant**	Error	0.35	0.10	0.18	0.52	0.33	0.13	0.12	0.70	0.28	0.12	0.08	0.57
		Correct	0.32	0.09	0.15	0.53	0.28	0.09	0.15	0.57	0.27	0.07	0.14	0.43
		ΔITPC	0.03	0.13	-0.22	0.29	0.05	0.16	-0.24	0.50	0.02	0.13	-0.18	0.41

Notes. HC = Healthy control group; BD = Bipolar spectrum disorder group; SZ = Schizophrenia spectrum disorder group; M = Mean; SD = Standard Deviation; Min = Minimum Value; Max = Maximum value; ITPC = Intertrial phase coherence; ΔERN, ΔPower, and ΔITPC = Error minus correct

trials for ERP amplitudes, theta power, and theta ITPC, respectively. All trials ERP amplitudes were computed including all trials in contrast with primary ERP analyses that included only correct trials matched to error trials.

Table S4. Descriptive statistics of behavioral performance measures on each flanker task for each group.

			HC				BD				SZ			
	N for Arrow / Unpleasant / Pleasant task		29 / 33 / 30				25 / 31 / 27				27 / 31 / 26			
**Task**	**Behavioral Measures**		*M*	*SD*	*Min*	*Max*	*M*	*SD*	*Min*	*Max*	*M*	*SD*	*Min*	*Max*
**Arrow**	**Accuracy**	Overall	90%	4%	80%	97%	88%	4%	80%	95%	88%	6%	75%	97%
		Congruent	97%	2%	91%	100%	96%	3%	87%	99%	96%	3%	87%	100%
		Incongruent	82%	8%	62%	95%	79%	7%	66%	91%	81%	10%	56%	95%
	**RT (s)**	Overall	0.43	0.05	0.36	0.58	0.48	0.09	0.34	0.69	0.48	0.09	0.36	0.70
		Congruent	0.40	0.04	0.34	0.53	0.45	0.08	0.33	0.65	0.45	0.08	0.34	0.62
		Incongruent	0.45	0.06	0.38	0.64	0.51	0.09	0.35	0.72	0.51	0.10	0.37	0.78
		Correct	0.43	0.05	0.37	0.59	0.49	0.08	0.36	0.67	0.49	0.08	0.36	0.69
		Error	0.36	0.04	0.29	0.44	0.40	0.09	0.25	0.74	0.40	0.09	0.29	0.73
		Post-correct Correct	0.43	0.05	0.36	0.59	0.49	0.08	0.36	0.68	0.49	0.08	0.36	0.68
		Post-correct Error	0.35	0.04	0.29	0.44	0.40	0.08	0.26	0.73	0.40	0.09	0.29	0.74
		Post-error Correct	0.44	0.06	0.38	0.63	0.50	0.09	0.35	0.71	0.50	0.09	0.38	0.74
		PES, Traditional	0.00	0.02	-0.03	0.04	0.01	0.02	-0.04	0.06	0.01	0.02	-0.03	0.06
		PES, Robust	0.02	0.03	-0.03	0.08	0.02	0.03	-0.03	0.09	0.03	0.03	0.00	0.14
**Unpleasant**	**Accuracy**	Overall	88%	4%	80%	96%	87%	5%	76%	97%	85%	6%	71%	95%
		Congruent	88%	4%	79%	95%	87%	6%	78%	98%	86%	6%	68%	95%
		Incongruent	88%	5%	79%	96%	86%	6%	75%	97%	85%	6%	73%	96%
	**RT (s)**	Overall	0.53	0.09	0.40	0.75	0.59	0.11	0.42	0.81	0.60	0.12	0.38	0.79
		Congruent	0.53	0.08	0.40	0.73	0.58	0.10	0.42	0.79	0.60	0.12	0.38	0.80
		Incongruent	0.54	0.09	0.41	0.77	0.59	0.11	0.43	0.83	0.61	0.12	0.39	0.81
		Correct	0.54	0.08	0.41	0.75	0.59	0.10	0.43	0.79	0.60	0.12	0.39	0.78
		Error	0.51	0.12	0.38	0.88	0.57	0.14	0.38	0.93	0.59	0.17	0.37	1.04
		Post-correct Correct	0.53	0.08	0.40	0.74	0.58	0.10	0.43	0.78	0.60	0.12	0.39	0.77
		Post-correct Error	0.51	0.11	0.38	0.88	0.57	0.14	0.39	0.94	0.59	0.17	0.36	1.01
		Post-error Correct	0.56	0.09	0.43	0.76	0.61	0.11	0.45	0.83	0.62	0.13	0.38	0.85
		PES, Traditional	0.03	0.03	-0.03	0.10	0.03	0.02	-0.03	0.07	0.02	0.04	-0.06	0.09
		PES, Robust	0.03	0.03	-0.02	0.10	0.03	0.03	-0.04	0.08	0.02	0.05	-0.07	0.10
**Pleasant**	**Accuracy**	Overall	89%	4%	78%	97%	88%	5%	76%	97%	85%	5%	72%	93%
		Congruent	89%	5%	77%	97%	88%	5%	78%	99%	86%	6%	72%	93%
		Incongruent	88%	4%	79%	97%	87%	6%	73%	97%	85%	5%	71%	93%
	**RT (s)**	Overall	0.50	0.07	0.40	0.66	0.54	0.11	0.42	0.87	0.57	0.12	0.39	0.86
		Congruent	0.49	0.07	0.40	0.66	0.54	0.10	0.42	0.85	0.56	0.12	0.40	0.87
		Incongruent	0.50	0.07	0.41	0.66	0.55	0.11	0.42	0.88	0.57	0.12	0.39	0.85
		Correct	0.50	0.07	0.41	0.66	0.55	0.10	0.43	0.83	0.57	0.12	0.40	0.83
		Error	0.46	0.07	0.36	0.63	0.52	0.15	0.34	1.03	0.55	0.18	0.36	1.19
		Post-correct Correct	0.50	0.07	0.40	0.66	0.54	0.10	0.43	0.83	0.57	0.12	0.40	0.83
		Post-correct Error	0.46	0.07	0.35	0.61	0.52	0.15	0.34	1.06	0.55	0.18	0.37	1.16
		Post-error Correct	0.52	0.08	0.41	0.68	0.56	0.10	0.43	0.84	0.59	0.12	0.41	0.82

PES, Traditional	0.02	0.03	-0.01	0.07	0.02	0.03	-0.05	0.07	0.02	0.03	-0.03	0.06
PES, Robust	0.03	0.03	-0.03	0.11	0.03	0.06	-0.04	0.23	0.02	0.04	-0.07	0.13

Notes. HC = Healthy control group; BD = Bipolar spectrum disorder group; SZ = Schizophrenia spectrum disorder group; M = Mean; SD = Standard Deviation; Min = Minimum Value; Max = Maximum value; RT = Reaction times; s = Seconds; PES = Post-error slowing. PES, Robust was calculated using a measure proposed by Derrfuss and colleagues (28). For all RT variables, trials were included in averaging if the trial RT was between 100 ms - 1500 ms and was within 3 SD of the median of that participants' trial-level RTs.

Table S5. Contrasts following two-way (Task × Response) analyses of covariance within each group (follow-up to Main Text **Table 1**).

Group	Type of contrast	Effects tested		EEG outcome		Contrast	Estimate	SE	df	*t*	*p*	
HC	Post-Hoc	Task		ERP		Arrow - Unpleasant	-2.77	0.62	163	-4.50	<.0001	***
						Arrow - Pleasant	-2.54	0.60	150	-4.27	<.0001	***
						Unpleasant - Pleasant	0.23	0.57	152	0.40	.6884	
		Task × Response interaction	Task effects within each Response	Theta ITPC	Error	Arrow - Unpleasant	0.14	0.03	156	5.42	<.0001	***
						Arrow - Pleasant	0.11	0.03	151	4.27	<.0001	***
						Unpleasant - Pleasant	-0.03	0.03	150	-1.11	.2686	
					Correct	Arrow - Unpleasant	0.02	0.03	156	0.86	.3903	
						Arrow - Pleasant	0.01	0.03	151	0.33	.7449	
						Unpleasant - Pleasant	-0.01	0.03	150	-0.54	.5891	
			Response effects within each Task	Theta ITPC	Arrow	Error - Correct	0.13	0.03	146	4.93	<.0001	***
					Unpleasant	Error - Correct	0.01	0.02	146	0.47	.6367	
					Pleasant	Error - Correct	0.03	0.03	146	1.01	.3149	
BD	Post-Hoc	Task		ERP		Arrow - Unpleasant	-3.17	0.71	153	-4.45	<.0001	***
						Arrow - Pleasant	-2.37	0.69	136	-3.42	.0008	**
						Unpleasant - Pleasant	0.80	0.67	143	1.20	.2340	
				Theta ITPC		Arrow - Unpleasant	0.09	0.02	152	4.35	<.0001	***
						Arrow - Pleasant	0.06	0.02	140	3.20	.0017	*
						Unpleasant - Pleasant	-0.02	0.02	143	-1.14	.2577	
SZ	Post-Hoc	Task		Theta ITPC		Arrow - Unpleasant	0.06	0.02	150	3.41	.0008	**
						Arrow - Pleasant	0.05	0.02	145	2.69	.0081	*
						Unpleasant - Pleasant	-0.01	0.02	140	-0.65	.5179	

Notes. SE = Standard error, df = Degrees of freedom; t = t-statistic; p = Statistical significance; ERP = Event-related potential; ITPC = Intertrial phase coherence; HC = Healthy control group; BD = Bipolar spectrum disorder group; SZ = Schizophrenia spectrum disorder group. * = $p < .01$; ** = $p < .001$; *** = $p < .0001$.

Table S6. Contrasts following two-way (Group × Response) analyses of covariance within each task (follow-up to Main Text **Table 2**).

Task	Type of contrast	Effects tested		EEG outcome		Contrast	Estimate	SE	df	*t*	*p*	
**Arrow**	**Planned**			**ERP**		HC vs. BD/SZ	0.94	1.97	76.00	0.48	.6355	
						BD vs. SZ	-1.90	1.15	76.00	-1.65	.1025	
				**Theta Power**		HC vs. BD/SZ	3.61	0.99	76.00	3.66	.0005	**
						BD vs. SZ	0.57	0.58	76.00	0.99	.3250	
				**Theta ITPC**		HC vs. BD/SZ	0.09	0.04	76.00	2.21	.0302	
						BD vs. SZ	0.02	0.02	76.00	1.05	.2963	
	**Post-Hoc**	**Group**		**Theta Power**		HC - BD	1.52	0.58	76.00	2.63	.0104	
						HC - SZ	2.09	0.56	76.00	3.71	.0004	**
						BD - SZ	0.57	0.58	76.00	0.99	.3250	
**Unpleasant**	**Planned**			**ERP**		HC vs. BD/SZ	1.34	1.64	90.00	0.82	.4155	
						BD vs. SZ	0.34	0.95	90.00	0.36	.7227	
				**Theta Power**		HC vs. BD/SZ	3.52	1.04	90.00	3.38	.0011	*
						BD vs. SZ	0.67	0.6	90.00	1.12	.2680	
				**Theta ITPC**		HC vs. BD/SZ	0.05	0.03	90.00	1.67	.0992	
						BD vs. SZ	0.00	0.02	90.00	-0.01	.9962	
	**Post-Hoc**	**Group × Response interaction**	**Group effects within each Response**	**Theta Power**	**Error**	HC - BD	1.88	0.63	110.00	3.00	.0034	*
						HC - SZ	2.80	0.64	109.00	4.36	<.0001	***
						BD - SZ	0.92	0.64	110.00	1.44	.1532	
					**Correct**	HC - BD	0.97	0.63	110.00	1.54	.1261	
						HC - SZ	1.40	0.64	109.00	2.18	.0314	
						BD - SZ	0.43	0.64	110.00	0.68	.5010	
			**Response effects within each Group**	**Theta Power**	**HC**	Error - Correct	3.28	0.28	92.00	11.80	<.0001	***
					**BD**	Error - Correct	2.37	0.29	92.00	8.25	<.0001	***
					**SZ**	Error - Correct	1.88	0.29	92.00	6.56	<.0001	***
**Pleasant**	**Planned**			**ERP**		HC vs. BD/SZ	2.23	1.75	78.00	1.27	.2065	
						BD vs. SZ	-0.07	1.05	78.00	-0.07	.9482	
				**Theta Power**		HC vs. BD/SZ	2.37	0.89	78.00	2.67	.0093	*
						BD vs. SZ	0.72	0.54	78.00	1.34	.1834	
				**Theta ITPC**		HC vs. BD/SZ	0.05	0.03	78.00	1.73	.0875	
						BD vs. SZ	0.01	0.02	78.00	0.51	.6138	
	**Post-hoc**			**Theta Power**	**Error**	HC - BD	0.99	0.55	103.00	1.81	.0734	
						HC - SZ	2.37	0.57	101.00	4.16	.0001	**

				BD - SZ	1.38	0.58	102.00	2.40	.0181	
**Response × Group interaction**	**Group effects within each response**		**Correct**	HC - BD	0.67	0.55	103.00	1.22	.2254	
				HC - SZ	0.72	0.57	101.00	1.26	.2100	
				BD - SZ	0.05	0.58	102.00	0.09	.9267	
	**Response effects within each group**	**Theta Power**	**HC**	Error - Correct	3.36	0.28	80.00	11.88	<.0001	***
			**BD**	Error - Correct	3.04	0.3	80.00	10.19	<.0001	***
			**SZ**	Error - Correct	1.70	0.3	80.00	5.61	<.0001	***

Notes. SE = Standard error, df = Degrees of freedom; *t* = *t*-statistic; *p* = Statistical significance; ERP = Event-related potential; ITPC = Intertrial phase coherence; HC = Healthy control group; BD = Bipolar spectrum disorder group; SZ = Schizophrenia spectrum disorder group. * = $p < .01$; ** = $p < .001$; *** = $p < .0001$.

Table S7. Three-way (Response × Group × Task) analyses of covariance results.

EEG Outcome	Predictor	SS	MS	Num df	Den df	*F*	*p*	η_p^2	
	Age	7.25	7.25	1	96.98	0.78	.3789	0.01	
	Accuracy	1.75	1.75	1	421.27	0.19	.6639	0.00	
	Response	3057.58	3057.58	1	406.15	329.60	<.0001	0.45	***
	Group	7.22	3.61	2	96.1	0.39	.6788	0.01	
**ERP**	Task	382.22	191.11	2	443.05	20.60	<.0001	0.09	***
	Response × Group	83.04	41.52	2	406.15	4.48	.0120	0.02	
	Response × Task	81.85	40.93	2	406.15	4.41	.0127	0.02	
	Group × Task	99.28	24.82	4	418.43	2.68	.0315	0.02	
	Response × Group × Task	4.11	1.03	4	406.15	0.11	.9787	0.00	
	Age	7.23	7.23	1	94.71	4.70	.0327	0.05	
	Accuracy	2.05	2.05	1	495.43	1.33	.2487	0.00	
	Response	981.30	981.30	1	403.59	638.30	<.0001	0.61	***
	Group	21.47	10.73	2	93.99	6.98	.0015	0.13	*
**Theta Power**	Task	15.63	7.81	2	430.4	5.08	.0066	0.02	*
	Response × Group	51.72	25.86	2	403.59	16.82	<.0001	0.08	***
	Response × Task	7.32	3.66	2	403.59	2.38	.0938	0.01	
	Group × Task	4.44	1.11	4	410.63	0.72	.5767	0.01	
	Response × Group × Task	3.69	0.92	4	403.59	0.60	.6628	0.01	
	Age	0.03	0.03	1	91.2	3.13	.0803	0.03	
	Accuracy	0.41	0.41	1	210.67	44.40	<.0001	0.17	***
	Response	0.18	0.18	1	405.93	18.98	<.0001	0.04	***
	Group	0.08	0.04	2	89.38	4.34	.0159	0.09	
**Theta ITPC**	Task	0.50	0.25	2	448.97	26.99	<.0001	0.11	***
	Response × Group	0.02	0.01	2	405.93	1.25	.2870	0.01	
	Response × Task	0.15	0.08	2	405.93	8.26	.0003	0.04	**
	Group × Task	0.01	0.00	4	432.21	0.34	.8535	0.00	
	Response × Group × Task	0.04	0.01	4	405.93	1.17	.3215	0.01	

Notes. ERP = Event-related potential; ITPC = Intertrial phase coherence; SS = Sum of squares; MS = Mean of squares; df = Degrees of freedom; *F* = *F*-statistic; *p* = Statistical significance; η_p^2 = Partial eta-squared; * = $p < .01$; ** = $p < .001$; *** = $p < .0001$.

Table S8. Correlations between EEG measures within each group.

S8.1. Within healthy control group correlations.

		Arrow						Unpleasant						Pleasant					
		ERN	CRN	Power E	Power C	ITPC E	ITPC C	ERN	CRN	Power E	Power C	ITPC E	ITPC C	ERN	CRN	Power E	Power C	ITPC E	ITPC C
**Arrow**	ERN																		
	CRN	**0.46**																	
	Power E	-0.11	-0.06																
	Power C	0.16	-0.37	0.36															
	ITPC E	-0.21	0.02	0.25	-0.31														
	ITPC C	-0.07	0.13	-0.05	-0.12	0.14													
**Unpleasant**	ERN	**0.55**	0.31	-0.12	-0.01	0.22	-0.05												
	CRN	0.38	**0.64**	0.01	-0.22	0.27	-0.04	**0.66**											
	Power E	-0.24	-0.11	**0.73**	0.41	0.03	0.08	-0.30	-0.08										
	Power C	0.05	-0.20	**0.48**	**0.79**	-0.23	0.01	-0.04	-0.12	**0.64**									
	ITPC E	-0.22	0.02	0.27	-0.09	0.22	-0.28	-0.23	0.07	0.38	-0.07								
	ITPC C	-0.14	-0.05	-0.06	-0.02	-0.01	**0.54**	-0.04	-0.02	0.04	-0.06	-0.27							
**Pleasant**	ERN	0.39	0.27	-0.17	-0.03	0.10	0.01	**0.78**	**0.46**	-0.08	0.13	-0.05	-0.21						
	CRN	0.27	**0.71**	-0.08	**-0.50**	0.31	0.06	**0.55**	**0.75**	-0.08	-0.26	0.07	-0.01	**0.57**					
	Power E	-0.05	0.05	**0.66**	0.06	0.20	0.13	-0.17	-0.04	**0.61**	0.22	0.16	-0.02	-0.15	0.11				
	Power C	0.11	-0.14	**0.48**	0.36	-0.09	0.14	-0.04	-0.04	**0.66**	**0.55**	-0.11	0.19	0.02	-0.03	**0.61**			
	ITPC E	-0.13	0.00	**0.55**	-0.06	**0.47**	-0.02	-0.13	0.05	0.40	0.01	0.31	0.07	-0.25	0.19	**0.57**	0.28		
	ITPC C	-0.06	0.04	-0.26	0.02	-0.21	0.16	-0.35	-0.43	-0.09	-0.01	-0.26	0.17	-0.08	-0.13	-0.01	0.10	0.09	

S8.2. Within bipolar spectrum disorder group correlations.

		Arrow						Unpleasant						Pleasant					
		ERN	CRN	Power E	Power C	ITPC E	ITPC C	ERN	CRN	Power E	Power C	ITPC E	ITPC C	ERN	CRN	Power E	Power C	ITPC E	ITPC C
**Arrow**	ERN																		
	CRN	**0.60**																	
	Power E	-0.38	0.04																
	Power C	-0.27	-0.14	**0.73**															
	ITPC E	0.09	0.41	**0.49**	0.31														
	ITPC C	-0.02	-0.25	0.05	0.45	-0.16													
**Unpleasant**	ERN	**0.48**	0.36	**-0.57**	**-0.78**	-0.01	-0.40												
	CRN	0.32	**0.61**	-0.02	-0.32	0.15	-0.24	0.45											
	Power E	-0.35	-0.01	**0.85**	**0.76**	**0.55**	0.08	-0.43	0.06										
	Power C	-0.30	-0.06	**0.60**	**0.77**	0.35	0.26	-0.35	-0.13	**0.83**									
	ITPC E	0.02	0.30	**0.50**	0.15	0.39	-0.08	-0.02	0.32	0.37	0.11								
	ITPC C	-0.07	-0.22	0.36	0.25	0.00	0.37	-0.01	0.01	0.37	0.34	0.23							
**Pleasant**	ERN	0.41	**0.60**	-0.29	**-0.52**	0.35	-0.32	**0.66**	0.25	-0.40	-0.29	-0.05	-0.33						

CRN	0.35	**0.70**	-0.04	-0.34	0.27	-0.32	**0.52**	**0.79**	0.10	0.02	0.25	-0.17	**0.53**				
Power E	-0.38	-0.04	**0.87**	**0.75**	**0.51**	0.04	**-0.50**	0.21	**0.93**	**0.72**	0.40	0.29	-0.39	0.15			
Power C	-0.31	-0.12	**0.73**	**0.85**	0.39	0.24	-0.44	-0.01	**0.82**	**0.83**	0.22	0.38	-0.29	-0.07	**0.79**		
ITPC E	-0.07	0.17	**0.57**	0.21	0.11	-0.21	-0.13	0.34	0.41	0.19	**0.55**	**0.47**	**-0.45**	0.13	**0.47**	0.27	
ITPC C	0.25	0.09	-0.01	0.29	0.16	**0.49**	-0.06	-0.01	0.27	0.26	**0.47**	0.20	-0.08	0.05	0.28	0.31	0.05

S8.3. Within schizophrenia spectrum disorder group correlations.

		**Arrow**						**Unpleasant**						**Pleasant**					
		ERN	CRN	Power E	Power C	ITPC E	ITPC C	ERN	CRN	Power E	Power C	ITPC E	ITPC C	ERN	CRN	Power E	Power C	ITPC E	ITPC C
**Arrow**	ERN																		
	CRN	**0.62**																	
	Power E	**-0.46**	-0.12																
	Power C	-0.32	-0.10	**0.71**															
	ITPC E	-0.42	-0.04	0.41	0.12														
	ITPC C	-0.16	-0.04	-0.17	-0.24	0.40													
**Unpleasant**	ERN	**0.55**	**0.53**	-0.32	0.00	-0.33	-0.08												
	CRN	0.42	**0.59**	-0.24	0.10	-0.10	0.01	**0.69**											
	Power E	**-0.49**	-0.10	**0.89**	**0.67**	**0.45**	-0.06	-0.30	-0.10										
	Power C	-0.41	-0.10	**0.79**	**0.65**	0.42	-0.08	-0.19	-0.04	**0.92**									
	ITPC E	-0.21	-0.03	0.10	0.35	0.01	0.17	0.01	0.25	0.32	0.23								
	ITPC C	-0.04	0.06	-0.32	-0.09	-0.22	-0.06	0.06	-0.08	-0.19	-0.21	0.05							
**Pleasant**	ERN	**0.69**	**0.59**	**-0.51**	-0.25	-0.39	0.17	**0.83**	**0.71**	**-0.47**	-0.36	-0.03	-0.10						
	CRN	0.39	**0.67**	-0.23	-0.02	0.09	0.28	**0.77**	**0.85**	-0.01	0.04	0.13	0.07	**0.70**					
	Power E	-0.29	-0.06	**0.80**	**0.57**	**0.55**	-0.14	-0.17	0.12	**0.89**	**0.85**	0.26	-0.30	-0.35	0.16				
	Power C	-0.24	-0.15	**0.75**	**0.68**	0.39	-0.21	-0.11	0.07	**0.82**	**0.89**	0.06	-0.28	-0.30	0.10	**0.88**			
	ITPC E	-0.22	-0.01	**0.49**	0.27	**0.70**	0.02	-0.15	-0.01	**0.61**	**0.46**	0.17	-0.07	-0.37	0.12	**0.61**	**0.50**		
	ITPC C	-0.08	0.04	-0.06	-0.37	0.12	0.26	-0.03	-0.19	0.08	0.08	-0.14	0.31	-0.24	0.01	0.05	-0.01	0.24	

Notes. For all S8 tables, Power-E = Error response theta power; Power-C = Correct response theta power; ITPC-E = Error response intertrial phase coherence; ITPC-C = Correct response intertrial phase coherence. All values reported are zero-order correlations in *r*. |*r*| larger than .45 are bolded and |*r*| larger than .70 are underlined.

Table S9. Intraclass correlation (ICC) of individual and task effects.

Effect	EEG Measure	Trial	HC	BD	SZ
Task	ERP	Error	0.16	0.22	0.02
		Correct	0.05	0.01	< 0.01
	Theta Power	Error	0.04	0.02	0.02
		Correct	< 0.01	< 0.01	< 0.01
	Theta ITPC	Error	0.36	0.20	0.25
		Correct	0.01	0.11	0.03
Participant	ERP	Error	0.49	0.37	0.61
		Correct	0.65	0.69	0.71
	Theta Power	Error	0.64	0.87	0.87
		Correct	0.59	0.80	0.76
	Theta ITPC	Error	0.24	0.30	0.23
		Correct	0.28	0.31	0.11

Notes. ERP = Event-related potential; ITPC = Intertrial phase coherence; HC = Healthy control group; BD = Bipolar spectrum disorder group; SZ = Schizophrenia spectrum disorder group. ICC was calculated for each EEG measure separately in each group (e.g., ERN in HC) with task valence and participant as the between-group effects, reflecting the contribution of the task and participant variances to each EEG measure.

Table S10. Correlations between EEG measures and behavioral flanker task performance within each group, and correlations with clinical measures for clinical groups only.

S10.1. Within healthy control group correlations.

			Arrow						Unpleasant						Pleasant					
			ERN	CRN	Power E	Power C	ITPC E	ITPC C	ERN	CRN	Power E	Power C	ITPC E	ITPC C	ERN	CRN	Power E	Power C	ITPC E	ITPC C
**Behavioral**	**Acc.**	Overall	0.04	0.06	-0.02	-0.06	0.04	0.29	-0.23	-0.03	0.21	-0.10	0.38	0.08	-0.11	0.00	0.07	0.00	0.28	0.42
		Congruent	-0.19	0.08	0.27	0.00	0.09	0.34	-0.20	-0.09	0.09	-0.15	0.16	0.24	-0.09	-0.03	0.00	0.06	0.17	0.40
		Incongruent	0.10	0.04	-0.11	-0.07	0.01	0.21	-0.21	0.04	0.27	-0.03	**0.50**	-0.10	-0.11	0.04	0.14	-0.07	0.37	0.36
	**RT**	Overall	0.06	0.10	-0.04	0.03	-0.23	0.20	-0.20	-0.40	-0.02	-0.18	0.16	0.01	-0.15	-0.42	0.03	0.00	0.02	0.33
		Congruent	0.06	0.06	-0.11	0.04	-0.24	0.25	-0.22	-0.42	-0.03	-0.18	0.15	0.00	-0.16	-0.42	0.06	0.00	0.03	0.33
		Incongruent	0.07	0.14	0.02	0.03	-0.22	0.16	-0.19	-0.37	-0.02	-0.17	0.17	0.01	-0.14	-0.42	0.01	-0.01	0.02	0.33
		Correct	0.07	0.11	-0.03	0.05	-0.25	0.19	-0.21	-0.39	-0.01	-0.16	0.17	-0.01	-0.15	-0.43	0.04	0.00	0.02	0.34
		Error	-0.01	0.07	-0.02	-0.08	-0.08	0.02	-0.12	-0.40	-0.14	-0.27	0.07	0.08	-0.18	-0.41	-0.06	-0.07	-0.03	0.15
		Post-correct Correct	0.08	0.13	-0.05	0.04	-0.26	0.19	-0.20	-0.38	-0.01	-0.17	0.17	0.00	-0.15	-0.42	0.05	0.00	0.03	0.35
		Post-correct Error	-0.02	0.09	-0.04	-0.09	-0.08	0.02	-0.11	-0.38	-0.15	-0.28	0.08	0.08	-0.16	-0.41	-0.05	-0.07	-0.03	0.16
		Post-error Correct	0.02	0.04	0.06	0.07	-0.23	0.20	-0.22	**-0.46**	0.01	-0.13	0.12	-0.03	-0.10	-0.39	0.00	-0.03	0.03	0.26
		PES, Traditional	-0.14	-0.19	0.27	0.11	-0.03	0.10	-0.12	-0.38	0.08	0.08	-0.12	-0.11	0.11	-0.08	-0.12	-0.10	0.03	-0.10
		PES, Robust	0.30	-0.01	0.22	0.15	-0.07	0.21	-0.17	-0.20	0.25	0.00	0.17	-0.30	-0.01	-0.12	-0.06	0.00	-0.11	-0.17

S10.2. Within bipolar spectrum disorder group correlations.

			Arrow						Unpleasant						Pleasant					
			ERN	CRN	Power E	Power C	ITPC E	ITPC C	ERN	CRN	Power E	Power C	ITPC E	ITPC C	ERN	CRN	Power E	Power C	ITPC E	ITPC C
**Behavioral**	**Acc.**	Overall	0.01	0.24	0.04	-0.08	0.25	-0.24	0.08	**0.47**	0.23	0.08	**0.59**	0.27	-0.09	0.17	0.35	0.12	**0.52**	0.34
		Congruent	-0.08	0.32	0.15	-0.22	0.28	-0.34	0.12	0.44	0.18	0.06	**0.57**	0.18	-0.11	0.22	0.27	0.02	**0.50**	0.35
		Incongruent	0.05	0.15	-0.02	0.00	0.19	-0.14	0.04	0.45	0.25	0.09	**0.54**	0.33	-0.06	0.08	0.36	0.20	0.45	0.27
	**RT**	Overall	0.10	0.11	-0.34	-0.16	-0.28	-0.12	-0.03	-0.28	-0.38	-0.22	-0.07	-0.01	-0.06	-0.17	-0.41	-0.23	-0.19	0.28
		Congruent	0.12	0.07	-0.35	-0.13	-0.33	-0.07	-0.03	-0.27	-0.37	-0.21	-0.06	0.00	-0.06	-0.17	-0.40	-0.22	-0.19	0.28
		Incongruent	0.09	0.14	-0.33	-0.18	-0.23	-0.17	-0.03	-0.30	-0.38	-0.22	-0.07	-0.01	-0.06	-0.16	-0.41	-0.24	-0.19	0.28
		Correct	0.12	0.11	-0.35	-0.16	-0.30	-0.10	-0.03	-0.28	-0.37	-0.21	-0.07	-0.01	-0.05	-0.17	-0.42	-0.24	-0.21	0.29
		Error	-0.03	0.06	-0.28	-0.14	-0.24	-0.24	-0.03	-0.35	-0.40	-0.19	-0.18	0.06	-0.06	-0.20	-0.40	-0.21	-0.19	0.20

	Post-correct Correct	0.12	0.12	-0.36	-0.17	-0.30	-0.10	-0.04	-0.27	-0.37	-0.22	-0.06	-0.01	-0.05	-0.16	-0.42	-0.24	-0.21	0.29
	Post-correct Error	-0.01	0.07	-0.29	-0.15	-0.24	-0.27	-0.03	-0.35	-0.40	-0.19	-0.17	0.07	-0.08	-0.20	-0.39	-0.21	-0.18	0.19
	Post-error Correct	0.12	0.02	-0.31	-0.08	-0.32	-0.04	-0.02	-0.24	-0.36	-0.18	-0.06	0.01	0.00	-0.16	-0.40	-0.21	-0.23	0.24
	PES, Traditional	0.03	-0.33	0.02	0.28	-0.21	0.24	0.08	0.07	-0.04	0.11	-0.01	0.08	0.20	-0.02	0.02	0.09	-0.11	-0.15
	PES, Robust	-0.41	**-0.57**	0.14	0.21	-0.05	0.28	0.02	0.12	0.07	0.03	0.22	0.04	0.03	-0.15	0.01	0.11	0.03	0.09
**Clinical**	HAMD-17	-0.15	-0.16	-0.29	-0.11	-0.19	0.08	-0.14	-0.08	-0.30	-0.39	0.02	-0.14	0.05	-0.18	-0.27	-0.16	-0.19	-0.02
	YMRS	0.04	0.30	-0.14	-0.35	0.08	-0.38	0.17	0.00	-0.06	-0.07	-0.06	0.20	0.36	0.15	-0.07	-0.06	-0.05	-0.09
	PANSS-positive	0.31	0.32	-0.07	-0.09	0.14	-0.24	0.07	-0.19	-0.07	-0.01	0.07	0.26	0.14	-0.07	-0.13	-0.03	0.21	-0.07
	PANSS-negative	-0.15	-0.32	-0.21	0.15	-0.14	0.27	-0.29	-0.01	-0.23	-0.23	0.10	-0.30	-0.10	-0.12	-0.13	0.01	-0.12	0.14
	PANSS-general	-0.21	-0.10	-0.17	-0.08	-0.07	0.04	0.02	-0.04	-0.21	-0.18	0.01	0.05	0.10	-0.05	-0.15	-0.01	-0.05	-0.05

S10.3. Within schizophrenia spectrum disorder group correlations.

			Arrow						Unpleasant						Pleasant					
			ERN	CRN	Power E	Power C	ITPC E	ITPC C	ERN	CRN	Power E	Power C	ITPC E	ITPC C	ERN	CRN	Power E	Power C	ITPC E	ITPC C
	**Acc.**	Overall	0.00	0.09	-0.22	-0.43	0.14	0.40	-0.21	-0.19	0.08	-0.12	0.27	0.37	-0.22	0.04	0.26	0.17	0.43	0.41
		Congruent	-0.01	0.04	0.05	-0.10	0.13	0.21	-0.16	-0.10	0.11	-0.11	0.26	0.30	-0.17	-0.01	0.25	0.17	0.43	0.26
		Incongruent	0.00	0.09	-0.27	**-0.45**	0.12	0.38	-0.24	-0.27	0.05	-0.12	0.25	0.42	-0.23	0.10	0.24	0.14	0.37	**0.52**
**Behavioral**	**RT**	Overall	-0.08	-0.35	**-0.48**	**-0.65**	-0.06	0.34	-0.23	**-0.46**	**-0.47**	**-0.49**	-0.34	0.22	-0.13	**-0.47**	**-0.72**	**-0.70**	-0.30	0.08
		Congruent	-0.09	-0.35	**-0.52**	**-0.68**	-0.01	0.36	-0.26	**-0.46**	**-0.47**	**-0.49**	-0.33	0.21	-0.14	**-0.48**	**-0.70**	**-0.68**	-0.28	0.08
		Incongruent	-0.07	-0.34	-0.44	**-0.61**	-0.10	0.31	-0.20	**-0.45**	**-0.47**	**-0.49**	-0.35	0.23	-0.12	**-0.45**	**-0.73**	**-0.72**	-0.31	0.07
		Correct	-0.11	-0.37	**-0.48**	**-0.64**	-0.07	0.34	-0.23	-0.44	**-0.49**	**-0.51**	-0.34	0.22	-0.12	**-0.47**	**-0.72**	**-0.70**	-0.31	0.08
		Error	0.06	-0.34	-0.43	**-0.53**	-0.14	0.12	-0.24	**-0.54**	-0.37	-0.37	-0.33	0.18	-0.13	-0.40	**-0.65**	**-0.60**	-0.24	0.07
		Post-correct Correct	-0.12	-0.37	**-0.48**	**-0.64**	-0.06	0.35	-0.23	-0.43	**-0.50**	**-0.53**	-0.33	0.22	-0.13	**-0.47**	**-0.71**	**-0.69**	-0.30	0.07
		Post-correct Error	0.05	-0.33	-0.40	**-0.51**	-0.13	0.11	-0.24	**-0.53**	-0.37	-0.37	-0.34	0.17	-0.11	-0.39	**-0.67**	**-0.62**	-0.25	0.04
		Post-error Correct	-0.02	-0.38	**-0.49**	**-0.65**	-0.14	0.26	-0.24	**-0.48**	-0.41	-0.42	-0.32	0.25	-0.07	-0.43	**-0.74**	**-0.74**	-0.33	0.14
		PES, Traditional	0.37	-0.09	-0.10	-0.12	-0.34	-0.32	-0.13	-0.37	0.10	0.14	-0.11	0.19	0.23	0.11	-0.29	-0.36	-0.19	0.32
		PES, Robust	0.01	-0.37	-0.35	-0.30	-0.23	0.11	-0.18	-0.26	0.19	0.27	-0.06	0.17	0.11	0.12	0.11	-0.07	-0.02	0.28
**Clinical**		HAMD-17	0.14	0.18	0.17	-0.01	0.05	-0.03	0.06	-0.06	0.39	0.28	-0.02	-0.05	-0.09	0.05	0.25	0.16	0.38	0.23
		YMRS	-0.06	0.21	-0.07	-0.35	0.27	0.19	-0.18	-0.04	0.03	-0.17	-0.09	0.15	-0.11	0.08	0.03	-0.17	0.35	0.25
		PANSS-positive	-0.08	0.04	-0.12	-0.37	0.35	0.27	-0.17	0.02	0.05	-0.07	-0.10	-0.12	-0.15	0.04	0.14	-0.02	0.35	0.28

	PANSS-negative	-0.13	-0.03	0.11	-0.24	0.21	0.45	-0.26	-0.31	-0.07	-0.14	-0.19	-0.11	-0.09	-0.31	-0.30	-0.34	-0.12	0.12
	PANSS-general	0.03	0.14	0.16	-0.07	0.11	0.19	0.05	0.03	0.31	0.17	-0.04	-0.10	0.04	0.07	0.13	0.03	0.33	0.08

Notes. For all S10 tables, Power-E = Error response theta power, Power-C = Correct response theta power, ITPC-E = Error response intertrial phase coherence, ITPC-C = Correct response intertrial phase coherence; Acc. = Accuracy; RT = Reaction time; PES = Post-error slowing; HAMD-17 = 17-item Hamilton Rating Scale for Depression; YMRS = Young Mania Rating Scale; PANSS = Positive and Negative Syndrome Scale. All values reported are zero-order correlations in *r* and |*r*| larger than .45 are bolded.

Table S11. Effects of antipsychotic medication on EEG measures

Group	EEG Outcome	Predictor	SS	MS	Num df	Den df	*F*	*p*	
SZ	ERP	Age	12.04	12.04	1	29.911	1.77	.1936	
		Accuracy	0.01	0.01	1	149	0.00	.9719	
		Response	462.62	462.62	1	125.56	67.98	<.0001	***
		Antipsychotic Use	1.85	1.85	1	27.991	0.27	.6063	
		Task	3.28	1.64	2	129.83	0.24	.7862	
		Response × Antipsychotic Use	20.79	20.79	1	125.56	3.06	.0829	
		Response × Task	16.24	8.12	2	125.56	1.19	.3066	
		Group × Task	7.12	3.56	2	126.64	0.52	.5942	
		Response × Antipsychotic use × Task	0.14	0.07	2	125.56	0.01	.9898	
	Theta Power	Age	4.76	4.76	1	28.369	3.50	.0719	
		Accuracy	0.27	0.27	1	153.34	0.20	.6591	
		Response	150.07	150.07	1	123.98	110.19	<.0001	***
		Antipsychotic Use	7.26	7.26	1	27.146	5.33	.0288	
		Task	6.95	3.48	2	127.12	2.55	.0819	
		Response × Antipsychotic Use	13.52	13.52	1	123.98	9.93	.0020	*
		Response × Task	3.85	1.92	2	123.98	1.41	.2474	
		Group × Task	4.17	2.08	2	124.72	1.53	.2207	
		Response × Antipsychotic use × Task	0.70	0.35	2	123.98	0.26	.7750	
	Theta ITPC	Age	0.02	0.02	1	28.318	2.26	.1440	
		Accuracy	0.13	0.13	1	75.971	17.59	<.0001	***
		Response	0.09	0.09	1	125.82	12.44	.0006	**
		Antipsychotic Use	0.00	0.00	1	24.442	0.16	.6899	
		Task	0.06	0.03	2	131.14	4.36	.0147	
		Response × Antipsychotic Use	0.07	0.07	1	125.82	9.99	.0020	*
		Response × Task	0.05	0.03	2	125.82	3.64	.0291	
		Group × Task	0.01	0.01	2	128.12	0.89	.4147	
		Response × Antipsychotic use × Task	0.03	0.01	2	125.82	2.00	.1402	
BD	ERP	Age	2.71	2.71	1	30.264	0.24	.6248	
		Accuracy	17.88	17.88	1	102.6	1.61	.2073	

	Response	1324.54	1324.54	1	123.85	119.30	<.0001	***
	Antipsychotic Use	25.09	25.09	1	31.471	2.26	.1428	
	Task	278.22	139.11	2	138.76	12.53	<.0001	***
	Response × Antipsychotic Use	23.30	23.30	1	123.85	2.10	.1500	
	Response × Task	18.96	9.48	2	123.85	0.85	.4282	
	Group × Task	45.38	22.69	2	129.92	2.04	.1337	
	Response × Antipsychotic use × Task	35.57	17.78	2	123.85	1.60	.2057	
**Theta Power**	Age	0.08	0.08	1	30.087	0.05	.8207	
	Accuracy	0.26	0.26	1	148.92	0.17	.6846	
	Response	316.39	316.39	1	122.7	200.27	<.0001	***
	Antipsychotic Use	7.87	7.87	1	30.731	4.98	.0330	
	Task	8.25	4.12	2	133.23	2.61	.0773	
	Response × Antipsychotic Use	4.80	4.80	1	122.7	3.04	.0840	
	Response × Task	3.50	1.75	2	122.7	1.11	.3337	
	Group × Task	1.81	0.90	2	125.62	0.57	.5662	
	Response × Antipsychotic use × Task	2.19	1.10	2	122.7	0.69	.5018	
**Theta ITPC**	Age	0.00	0.00	1	27.617	0.10	.7516	
	Accuracy	0.12	0.12	1	63.392	11.75	.0011	*
	Response	0.03	0.03	1	123.2	3.16	.0777	
	Antipsychotic Use	0.01	0.01	1	28.932	0.72	.4043	
	Task	0.19	0.10	2	139.15	9.28	.0002	**
	Response × Antipsychotic Use	0.00	0.00	1	123.2	0.01	.9350	
	Response × Task	0.04	0.02	2	123.2	1.74	.1792	
	Group × Task	0.00	0.00	2	133.75	0.02	.9821	
	Response × Antipsychotic use × Task	0.00	0.00	2	123.2	0.07	.9288	

ERP = Event-related potential; ITPC = Intertrial phase coherence; HC = Healthy control group; BD = Bipolar spectrum disorder group; SZ = Schizophrenia spectrum disorder group; SS = Sum of squares; MS = Mean of squares; *F* = *F*-statistic; df = Degrees of freedom; *p* = Statistical significance; * = $p < .01$; ** = $p < .001$; *** = $p < .0001$.

Table S12. Descriptive statistics of participants' self-reported affect of the pleasantness, unpleasantness, and excitability of each flanker task.

		HC				BD				SZ			
N for Arrow / Unpleasant / Pleasant task		28 / 28 / 27				33 / 32 / 33				31 / 31 / 29			
	Affect Self-Rating	*M*	*SD*	*Min*	*Max*	*M*	*SD*	*Min*	*Max*	*M*	*SD*	*Min*	*Max*
Arrow	Pleasant	64.36	23.73	10	100	53.55	21.68	20	100	49.84	28.71	0	100
	Unpleasant	24.86	24.55	0	80	38.64	26.03	0	90	38.23	26.97	0	100
	Excited	38.71	26.61	0	85	42.97	25	10	100	34.19	27.24	0	90
Unpleasant	Pleasant	45.89	32.18	0	100	32.28	26.01	0	100	35.65	29.99	0	100
	Unpleasant	48.04	33.04	0	100	57.12	25.62	0	100	55.32	28.78	0	100
	Excited	38.11	24.25	0	90	43.81	24.37	0	90	41.42	27.91	0	90
Pleasant	Pleasant	69.07	22.83	20	100	56.48	26.71	0	100	53.62	28.78	10	100
	Unpleasant	22.78	23.75	0	80	38.55	27.3	0	100	32.76	23.78	0	75
	Excited	34.78	26.47	0	75	40.33	22.92	0	80	33.79	29.45	0	100

Notes. Ratings were made on a scale of 0 to 100. HC = Healthy control group; BD = Bipolar spectrum disorder group; SZ = Schizophrenia spectrum disorder group; M = Mean; SD = Standard Deviation; Min = Minimum Value; Max = Maximum value.

Table S13. One-way analyses of covariance results comparing the three ratings (feeling pleasant, feeling unpleasant, feeling excited) across all three tasks (arrow, unpleasant, pleasant), separately within each diagnostic group.

Table S13.1. One-way analysis of variance of the effect of task on self-ratings.

Group	Type of Rating	*F*	*df*	*p*	
HC	Unpleasant	7.23	2, 80	.0013	**
	Pleasant	5.87	2, 80	.0042	**
	Excited	0.18	2, 80	.8318	
BD	Unpleasant	5.34	2, 95	.0064	**
	Pleasant	9.11	2, 95	.0002	***
	Excited	0.19	2, 95	.8312	
SZ	Unpleasant	5.92	2, 88	.0039	**
	Pleasant	3.21	2, 88	.0452	
	Excited	0.71	2, 88	.4946	

Table S13.2. Contrasts following one-way analyses of variance.

Group	Type of Rating	Contrast	Estimate	SE	df	*t*	*p*	
HC	Unpleasant	Arrow - Unpleasant	-23.18	7.34	80	-3.16	.0023	*
		Arrow - Pleasant	2.08	7.41	80	0.28	.7798	
		Unpleasant - Pleasant	25.26	7.41	80	3.41	.0010	*
	Pleasant	Arrow - Unpleasant	18.46	7.12	80	2.60	.0113	
		Arrow - Pleasant	-4.72	7.18	80	-0.66	.5132	
		Unpleasant - Pleasant	-23.18	7.18	80	-3.23	.0018	*
BD	Unpleasant	Arrow - Unpleasant	-18.49	6.53	95	-2.83	.0057	*
		Arrow - Pleasant	0.09	6.48	95	0.01	.9888	
		Unpleasant - Pleasant	18.58	6.53	95	2.84	.0055	*
	Pleasant	Arrow - Unpleasant	21.26	6.17	95	3.44	.0009	**
		Arrow - Pleasant	-2.94	6.13	95	-0.48	.6325	
		Unpleasant - Pleasant	-24.20	6.17	95	-3.92	.0002	**
SZ	Unpleasant	Arrow - Unpleasant	-17.10	6.77	88	-2.53	.0133	
		Arrow - Pleasant	5.47	6.89	88	0.79	.4293	
		Unpleasant - Pleasant	22.56	6.89	88	3.28	.0015	*

Notes. F = *F*-statistic; df = Degrees of freedom *p* = Statistical significance; SE = Standard error, *t* = *t*-statistic; HC = Healthy control group; BD = Bipolar spectrum disorder group; SZ = Schizophrenia spectrum disorder group; * = $p < .01$; ** = $p < .001$; *** = $p < .0001$.

Table S14. Mood state of participants in each group.

	Euthymic (HAMD-17 and YMRS both < 8)	Depressed (HAMD-17 $\geq$ 8)	Manic or Hypomanic (YMRS $\geq$ 8)	Mixed (HAMD-17 and YMRS both $\geq$ 8)
HC	33	0	0	0
BD	19	12	7	5
SZ	16	11	8	3

Notes. Based on the cutoff scores used, some participants are included in the count for either (a) depressed and mixed mood states or (b) manic/hypomanic and mixed mood states. HAMD-17 = 17-item Hamilton Rating Scale for Depression; YMRS = Young Mania Rating Scale; HC = Healthy control group; BD = Bipolar spectrum disorder group; SZ = Schizophrenia spectrum disorder group.

Table S15. Analyses of early-baseline event-related potentials (ERPs). Three-way (Response × Group × Task) analyses of covariance results for early-baseline ERPs and two-way analyses of covariance results (Response × Task) within the healthy control (HC) group.

	Predictor	SS	MS	Num df	Den df	*F*	*p*	
**All participants**	Age	2.50	2.50	1	95.12	0.27	.6040	
	Accuracy	7.80	7.80	1	472.84	0.83	.3635	
	Response	4388.90	4388.90	1	404.03	466.78	<.0001	***
	Group	24.50	12.20	2	94.31	1.30	.2772	
	Task	52.50	26.30	2	436.18	2.79	.0623	
	Response × Group	61.30	30.60	2	404.03	3.26	.0395	
	Response × Task	63.70	31.80	2	404.03	3.39	.0348	
	Group × Task	109.80	27.40	4	413.35	2.92	.0211	
	Response × Group × Task	42.30	10.60	4	404.03	1.13	.3441	
**Within HC group**	Age	4.92	4.92	1	33.891	0.53	.4697	
	Accuracy	0.13	0.13	1	175.416	0.01	.9061	
	Response	1900.03	1900.03	1	145.283	206.56	<.0001	***
	Task	10.32	5.16	2	152.447	0.56	.5718	
	Response × Task	19.16	9.58	2	145.283	1.04	.3555	

Notes. SS = Sum of squares; MS = Mean of squares; F = F-statistic; df = Degrees of freedom; p = Statistical significance; * = $p < .01$; ** = $p < .001$; *** = $p < .0001$. Early-baseline ERPs were computed using baseline of -400 to -200 ms and were also computed including all trials.

Table S16. Cluster Analysis Results

Table S16.1. Principal component analysis, rotations by component

Task	EEG Measure	Trial	Component 1	Component 2	Component 3
Arrow	Theta Power	Error	-0.384	-0.134	-0.017
		Correct	-0.311	0.070	-0.404
	Theta ITPC	Error	-0.233	-0.104	0.398
		Correct	-0.045	0.574	0.026
Unpleasant	Theta Power	Error	-0.409	-0.028	-0.040
		Correct	-0.356	0.035	-0.326
	Theta ITPC	Error	-0.161	-0.254	0.446
		Correct	-0.052	0.563	0.198
Pleasant	Theta Power	Error	-0.392	-0.043	0.037
		Correct	-0.374	0.068	-0.213
	Theta ITPC	Error	-0.271	-0.069	0.482
		Correct	-0.099	0.492	0.230
Variance Explained			45.2%	14.0%	11.7%

Table S16.2. Crosstab tables on distribution of clusters from 2-cluster, 3-cluster, and diagnostic groups

		Diagnostic			3-Cluster		
	Cluster	HC	BD	SZ	1	2	3
2-Cluster	1	1	7	12	0	0	20
	2	27	14	11	35	17	0
3-Cluster	1	15	10	10			
	2	12	4	1			
	3	1	7	12			

Table S16.3. Descriptive statistics and ANOVA results on clinical measures by clusters

		Cluster 1					Cluster 2													
		N	*M*	*SD*	*Min*	*Max*	*N*	*M*	*SD*	*Min*	*Max*						t or χ^2	*df*	*p*	
2-Cluster Solution	**HAMD-17**	20	7.75	6.29	0	19	52	3.48	5.20	0	20						2.94	70	0.0045	1 > 2
	**YMRS**	20	4.70	4.24	0	16	52	2.33	3.80	0	17						2.30	70	0.0245	
	**PANSS-Positive**	20	11.35	4.23	7	21	52	8.87	3.33	7	23						2.63	70	0.0106	
	**PANSS-Negative**	20	10.90	5.33	7	24	51	8.27	3.03	7	19						2.61	69	0.0110	
	**PANSS-General**	20	24.45	5.60	16	32	52	19.58	6.08	16	40						3.11	70	0.0027	1 > 2
	**With Psychosis**	20	80%				52	38%									8.38	1	0.0038	1 > 2
		Cluster 3					Cluster 1					Cluster 2								
		N	*M*	*SD*	*Min*	*Max*	*N*	*M*	*SD*	*Min*	*Max*	*N*	*M*	*SD*	*Min*	*Max*	F or χ^2	*df*	*p*	
3-Cluster Solution	**HAMD-17**	20	7.75	6.29	0	19	35	3.71	5.13	0	20	17	3.00	5.49	0	19	4.37	2, 69	0.0164	
	**YMRS**	20	4.70	4.24	0	16	35	2.86	4.33	0	17	17	1.24	2.08	0	6	3.67	2, 69	0.0306	
	**PANSS-Positive**	20	11.35	4.23	7	21	35	9.43	3.82	7	23	17	7.71	1.45	7	12	4.88	2, 69	0.0104	
	**PANSS-Negative**	20	10.90	5.33	7	24	35	8.06	2.60	7	18	16	8.75	3.87	7	19	3.57	2, 68	0.0337	
	**PANSS-General**	20	24.45	5.60	16	32	35	20.00	6.38	16	40	17	18.71	5.47	16	33	5.08	2, 69	0.0088	3 > 1 = 2
	**With Psychosis**	20	80%				35	43%				17	29%				10.80	1	0.0045	3 > 2

Notes. Analyses were based on 72 complete cases. EEG = Electroencephalogram; ITPC = Intertrial phase coherence. Parallel analysis suggested 2 components to be extracted.

Table S17. Analyses of event-related potentials (ERPs) including all trials within each task and each group.

S17.1. Two-way analyses of covariance results for ERPs including all trials within each task (Response × Group) and within each group (Response × Task).

	Task or Group	Predictor	SS	MS	Num df	Den df	*F*	*p*	
**Within Task Analyses**	Arrow	Age	27.12	27.12	1	76.00	2.40	.1259	
		Accuracy	28.78	28.78	1	76.00	2.54	.1150	
		Response	878.85	878.85	1	78.00	77.62	<.0001	***
		Group	91.21	45.61	2	76.00	4.03	.0217	
		Response × Group	17.61	8.80	2	78.00	0.78	.4631	
	Unpleasant	Age	33.55	33.55	1	90.00	6.22	.0145	
		Accuracy	0.09	0.09	1	90.00	0.02	.9000	
		Response	701.93	701.93	1	92.00	130.07	<.0001	***
		Group	7.80	3.90	2	90.00	0.72	.4881	
		Response × Group	29.65	14.83	2	92.00	2.75	.0694	
	Pleasant	Age	34.33	34.33	1	78.00	4.50	.0371	
		Accuracy	0.66	0.66	1	78.00	0.09	.7692	
		Response	758.09	758.09	1	80.00	99.33	<.0001	***
		Group	21.53	10.76	2	78.00	1.41	.2502	
		Response × Group	34.20	17.10	2	80.00	2.24	.1130	
**Within Group Analyses**	HC	Age	4.41	4.41	1	34.84	0.49	.4868	
		Accuracy	0.30	0.30	1	150.55	0.03	.8548	
		Response	1119.79	1119.79	1	145.13	125.52	<.0001	***
		Task	329.19	164.59	2	155.31	18.45	<.0001	***
		Response × Task	6.24	3.12	2	145.13	0.35	.7056	
	BD	Age	0.07	0.07	1	30.03	0.01	.9300	
		Accuracy	0.45	0.45	1	107.76	0.05	.8180	
		Response	882.30	882.30	1	128.18	104.04	<.0001	***
		Task	353.07	176.54	2	144.68	20.82	<.0001	***
		Response × Task	8.74	4.37	2	128.18	0.52	.5985	
	SZ	Age	21.33	21.33	1	31.05	3.46	.0725	
		Accuracy	7.90	7.90	1	150.58	1.28	.2597	

Response	434.11	434.11	1	130.75	70.35	<.0001	***
Task	18.50	9.25	2	140.32	1.50	.2269	
Response × Task	9.48	4.74	2	130.75	0.77	.4660	

S17.2. Contrasts following two-way analyses of covariance for ERPs including all trials.

	Task or Group	Type of contrast	Effect Tested	Contrast	Estimate	SE	df	*t*	*p*	
**Within Task Analyses**	**Arrow**	**Planned**	**Group**	HC vs. BD/SZ	1.79	1.68	76	1.07	.2885	
				BD vs. SZ	-2.60	0.98	76	-2.66	.0095	*
	**Unpleasant**	**Planned**	**Group**	HC vs. BD/SZ	1.77	1.48	90	1.20	.2350	
				BD vs. SZ	0.14	0.86	90	0.16	.8712	
	**Pleasant**	**Planned**	**Group**	HC vs. BD/SZ	2.70	1.61	78	1.68	.0972	
				BD vs. SZ	0.04	0.97	78	0.05	.9637	
**Within Group Analyses**	**HC**	**Post-Hoc**	**Task**	Arrow - Unpleasant	-3.07	0.59	163	-5.23	<.0001	***
				Arrow - Pleasant	-3.11	0.57	151	-5.45	<.0001	***
				Unpleasant - Pleasant	-0.03	0.55	153	-0.06	.9554	
	**BD**	**Post-Hoc**	**Task**	Arrow - Unpleasant	-3.61	0.62	153	-5.87	<.0001	***
				Arrow - Pleasant	-3.23	0.60	136	-5.42	<.0001	***
				Unpleasant - Pleasant	0.38	0.58	143	0.65	.5150	

Notes. SS = Sum of squares; MS = Mean of squares; *F* = *F*-statistic; df = Degrees of freedom; *p* = Statistical significance; SE = Standard error; *t* = *t*-statistic; * = $p < .01$; ** = $p < .001$; *** = $p < .0001$. These ERPs were computed including all trials in contrast with primary ERP analyses that included only correct trials matched to error trials.

Table S18. Root mean square of the standardized measurement error (RMS(SME)) values and descriptive statistics of signal-to-noise (SNR) ratio.

Task	EEG Measure	Trial	N	RMS(SME)	SNR Mean	SNR SD	SNR Median
Arrow	Theta Power	Error	81	0.70	6.68	4.99	6.72
		Correct	81	0.75	1.48	2.90	1.76
	ERP	Error	81	1.73	-0.63	3.23	-0.25
		Correct	81	1.65	3.57	3.71	3.17
		All Correct	81	0.56	6.73	7.38	6.62
Unpleasant	Theta Power	Error	95	0.74	4.82	4.82	5.11
		Correct	95	0.74	1.06	3.72	1.78
	ERP	Error	95	1.77	1.30	2.55	0.78
		Correct	95	1.67	4.27	3.50	3.31
		All Correct	95	0.58	10.16	6.81	8.80
Pleasant	Theta Power	Error	83	0.74	5.72	4.49	6.03
		Correct	83	0.69	1.34	3.47	1.82
	ERP	Error	83	1.71	1.10	2.51	0.97
		Correct	83	1.66	3.98	3.12	3.62
		All Correct	83	0.57	10.57	6.72	10.29

Notes. ERP = Event-related potential; SD = Standard deviation.

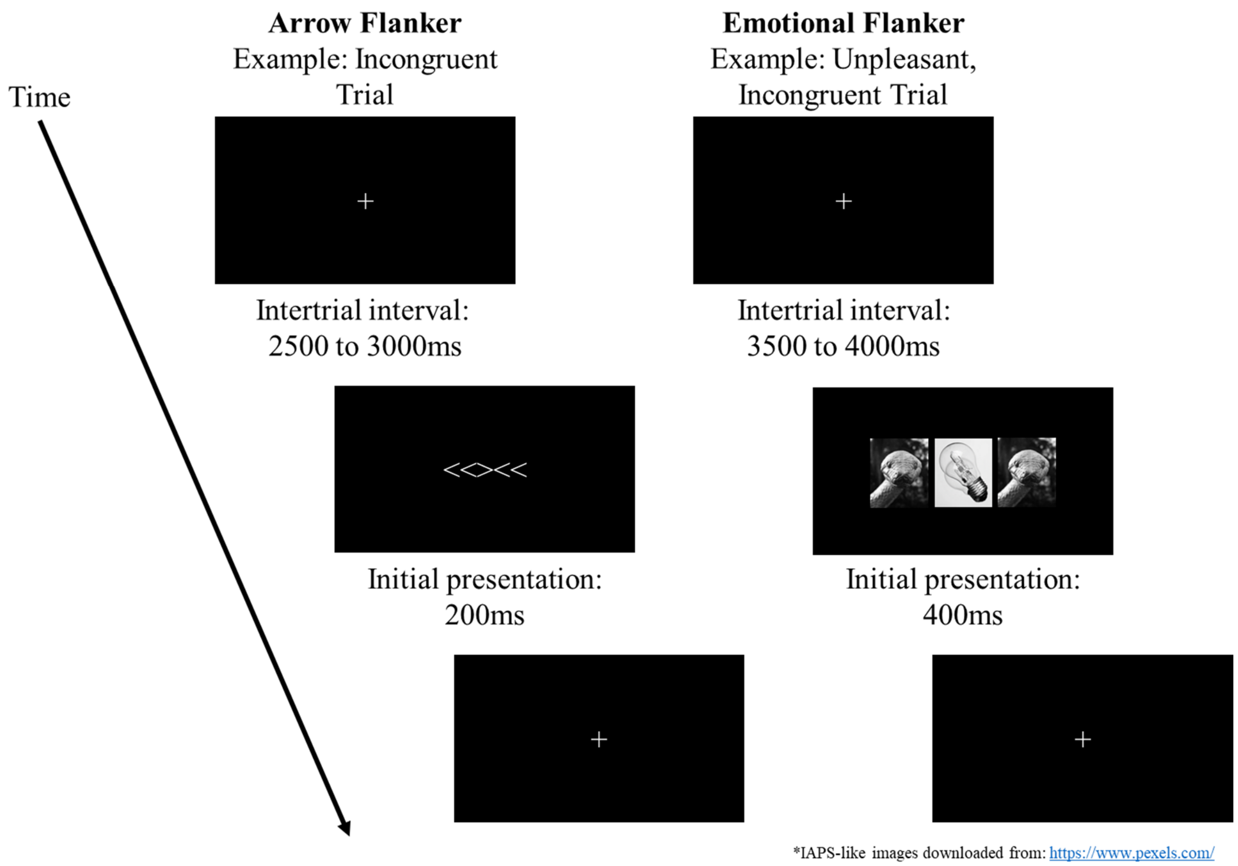

Figure S1. Visual representation of the Arrow task with incongruent trials as examples (left) and the Unpleasant task with a neutral target and unpleasant flanker stimuli (right) are presented. The Pleasant task has the exact same structure as the Unpleasant task, except pleasant images are used instead of unpleasant images.

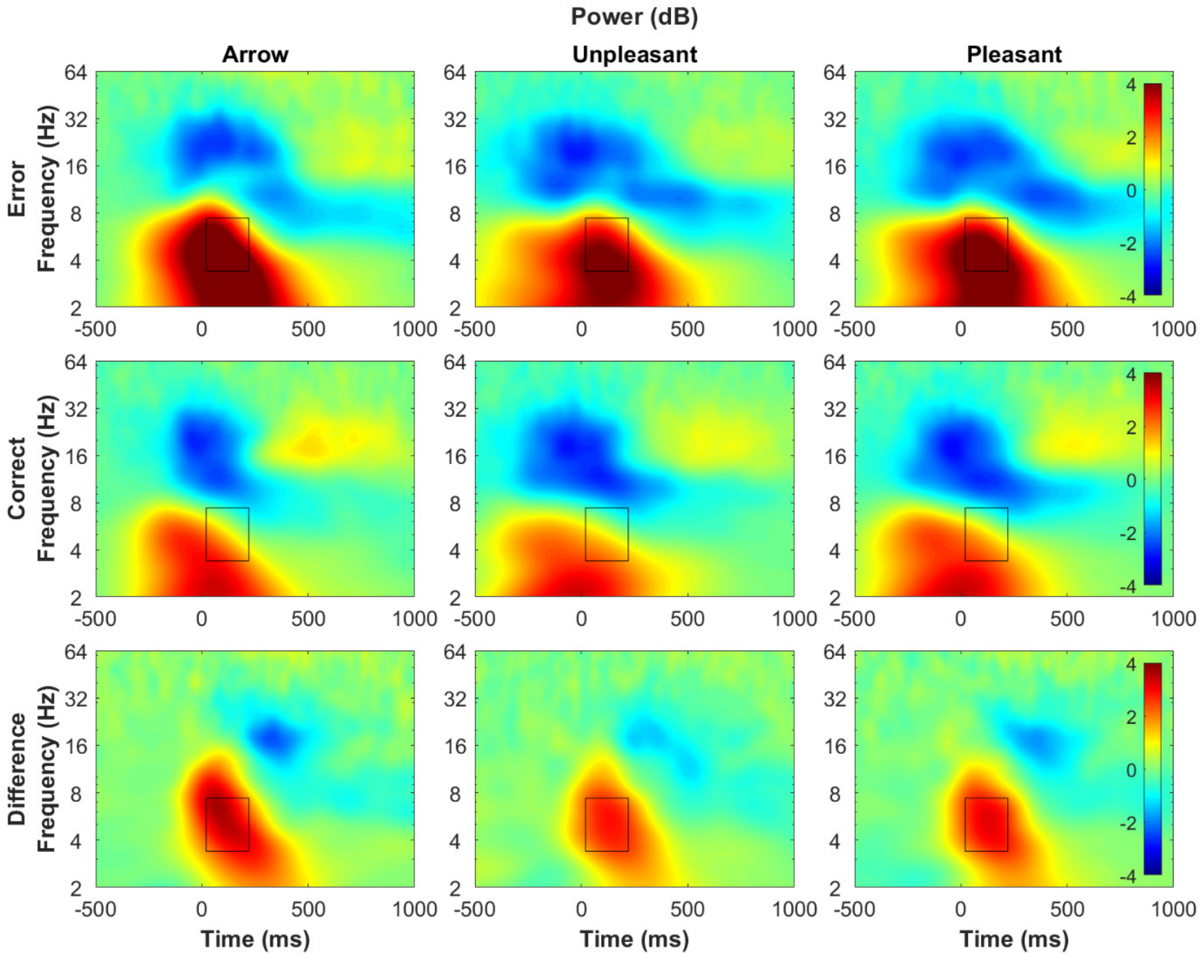

Figure S2. Time-frequency decomposition plots of total power at Cz electrode. Rows: Top = Error responses; Middle = Correct responses; Bottom = Difference between error and correct responses (error minus correct), collapsing all three groups (healthy control, bipolar disorder, schizophrenia) to visualize time-frequency power differences between correct and error responses. Columns: Left = Arrow task; Middle = Unpleasant task; Right = Pleasant task. Horizontal axis reflects time (-500ms to 1000ms relative to response; 0ms indicates when response was made) and vertical axis represents the frequency (2 to 64 Hz, logarithmically spaced). Heatmap is scaled from -4 (blue) to 4 (red) decibel (dB) for all plots for comparability. The boxed area represents the area that theta-band data was extracted from (20 to 220 ms and 3.38 to 7.38 Hz).

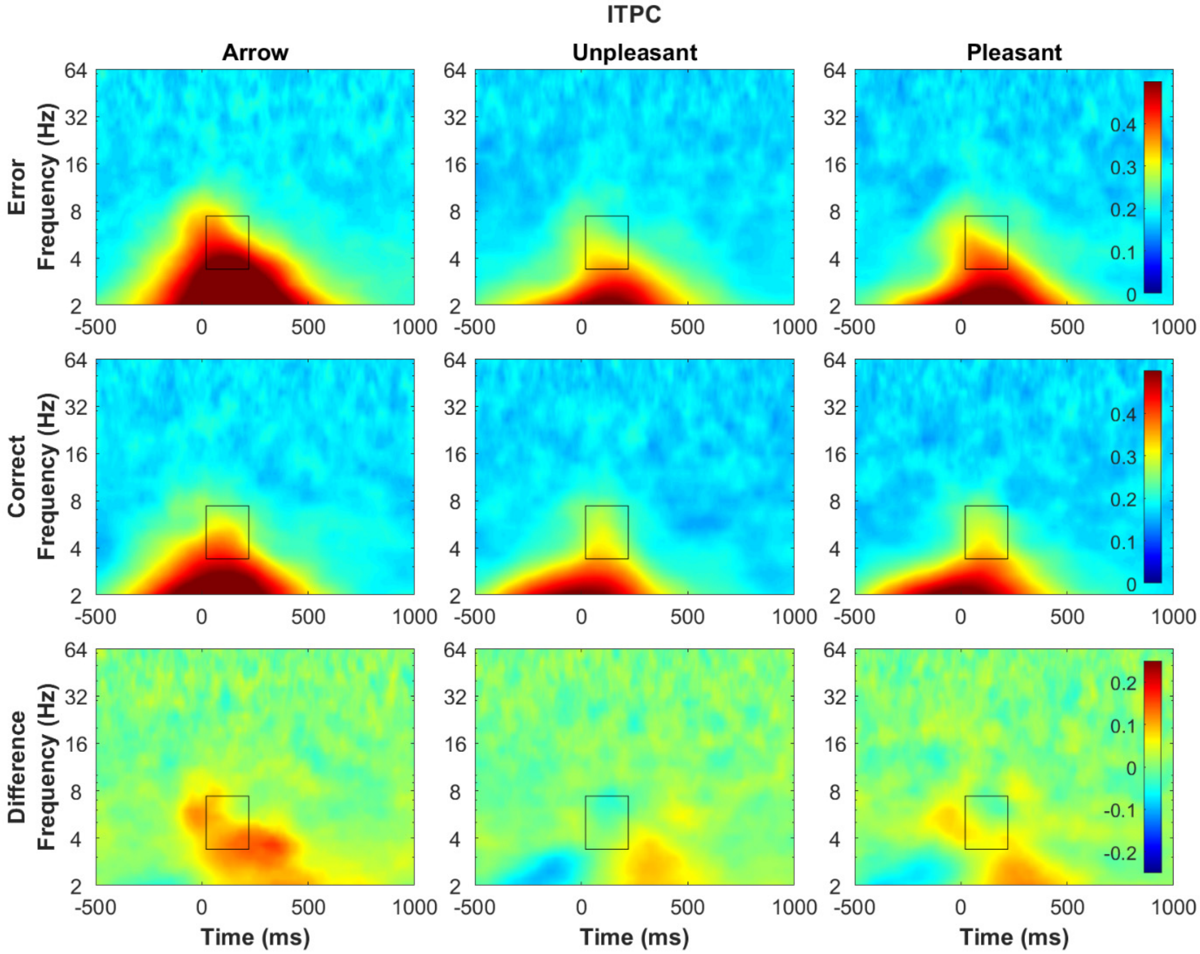

Figure S3. Time-frequency decomposition plots of intertrial phase coherence (ITPC) at Cz electrode. Rows: Top = Error responses; Middle = Correct responses; Bottom = Difference between error and correct responses (error minus correct), collapsing all three groups (healthy control, bipolar spectrum disorder group, schizophrenia spectrum disorder group) to visualize time-frequency power differences between correct and error responses. Columns: Left = Arrow task; Middle = Unpleasant task; Right = Pleasant task. Horizontal axis reflects time (-500ms to 1000ms relative to response; 0ms indicates when response was made) and vertical axis represents the frequency (2 to 64 Hz, logarithmically spaced). Heatmap is scaled for 0 (blue) to 0.5 (red) for the error and correct responses and -0.25 to 0.25 for the difference. The boxed area represents the area that theta-band data was extracted from (20 to 220 ms and 3.38 to 7.38 Hz).

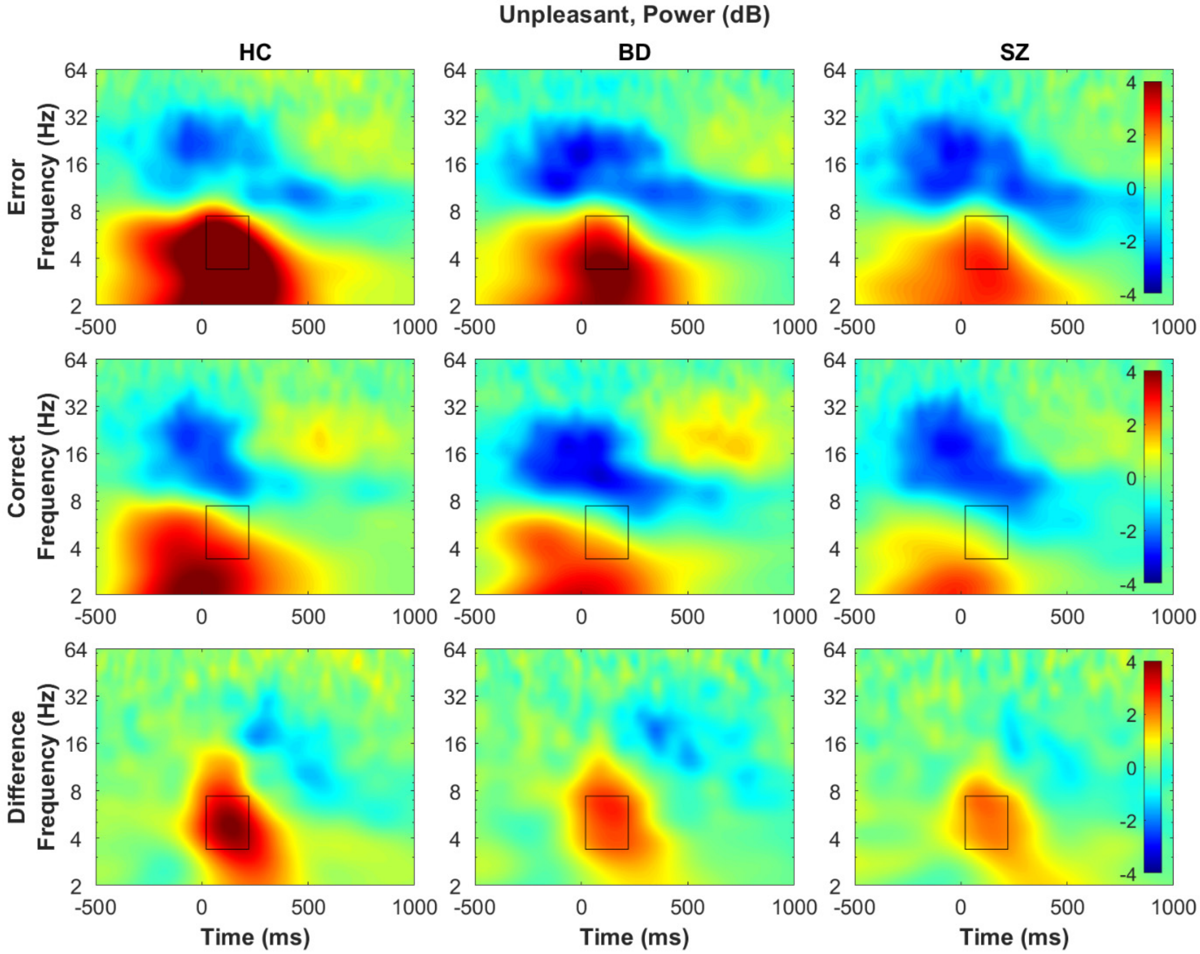

Figure S4. Time-frequency decomposition plots of power at Cz electrode for the Unpleasant task. Rows: Top = Error responses; Middle = Correct responses; Bottom = Difference between error and correct responses (error minus correct). Columns: Left = Healthy control group (HC); Middle = Bipolar spectrum disorder group (BD); Right = Schizophrenia spectrum disorder group (SZ). Horizontal axis reflects time (-500ms to 1000ms relative to response; 0ms indicates when response was made) and vertical axis represents the frequency (2 to 64 Hz, logarithmically spaced). Heatmap is scaled from -4 (blue) to 4 (red) decibel (dB) for all plots for comparability. The boxed area represents the area that theta-band data was extracted from (20 to 220 ms and 3.38 to 7.38 Hz). Results (Tables 2 & S6): There is a Response × Group interaction effect, such that Error theta power in the HC group (top left) was larger than Error theta power in BD and SZ groups (top middle and right), but BD and SZ groups were comparable. Correct theta power was comparable across all groups (middle row). In all groups, Error theta power was larger than Correct theta power (bottom row).

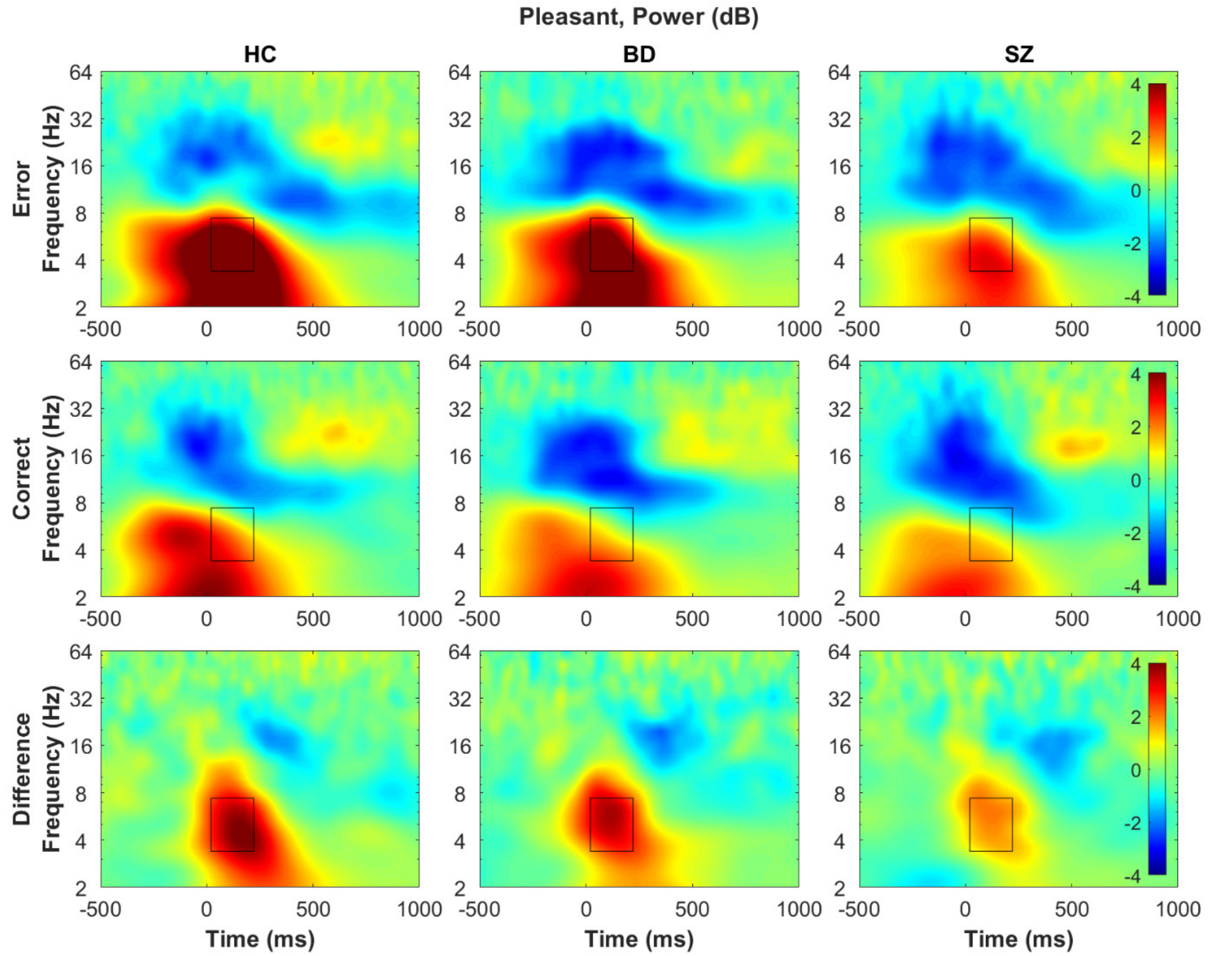

Figure S5. Time-frequency decomposition plots of power at Cz electrode for the Pleasant task. Rows: Top = Error responses; Middle = Correct responses; Bottom = Difference between error and correct responses (error minus correct). Columns: Left = Healthy control group (HC); Middle = Bipolar spectrum disorder group (BD); Right = Schizophrenia spectrum disorder group (SZ). Horizontal axis reflects time (-500ms to 1000ms relative to response; 0ms indicates when response was made) and vertical axis represents the frequency (2 to 64 Hz, logarithmically spaced). Heatmap is scaled from -4 (blue) to 4 (red) decibel (dB) for all plots for comparability. The boxed area represents the area that theta-band data was extracted from (20 to 220 ms and 3.38 to 7.38 Hz). Results (Tables 2 & S6): There was a Response × Group interaction effect such that the Error theta power in the HC group (top left) was larger than Error theta power in the SZ group (top right), but Error theta power in BD (top middle) was comparable to both. Correct theta power (middle row) were comparable across all groups. In all groups, Error theta power was larger than Correct theta power (bottom row).

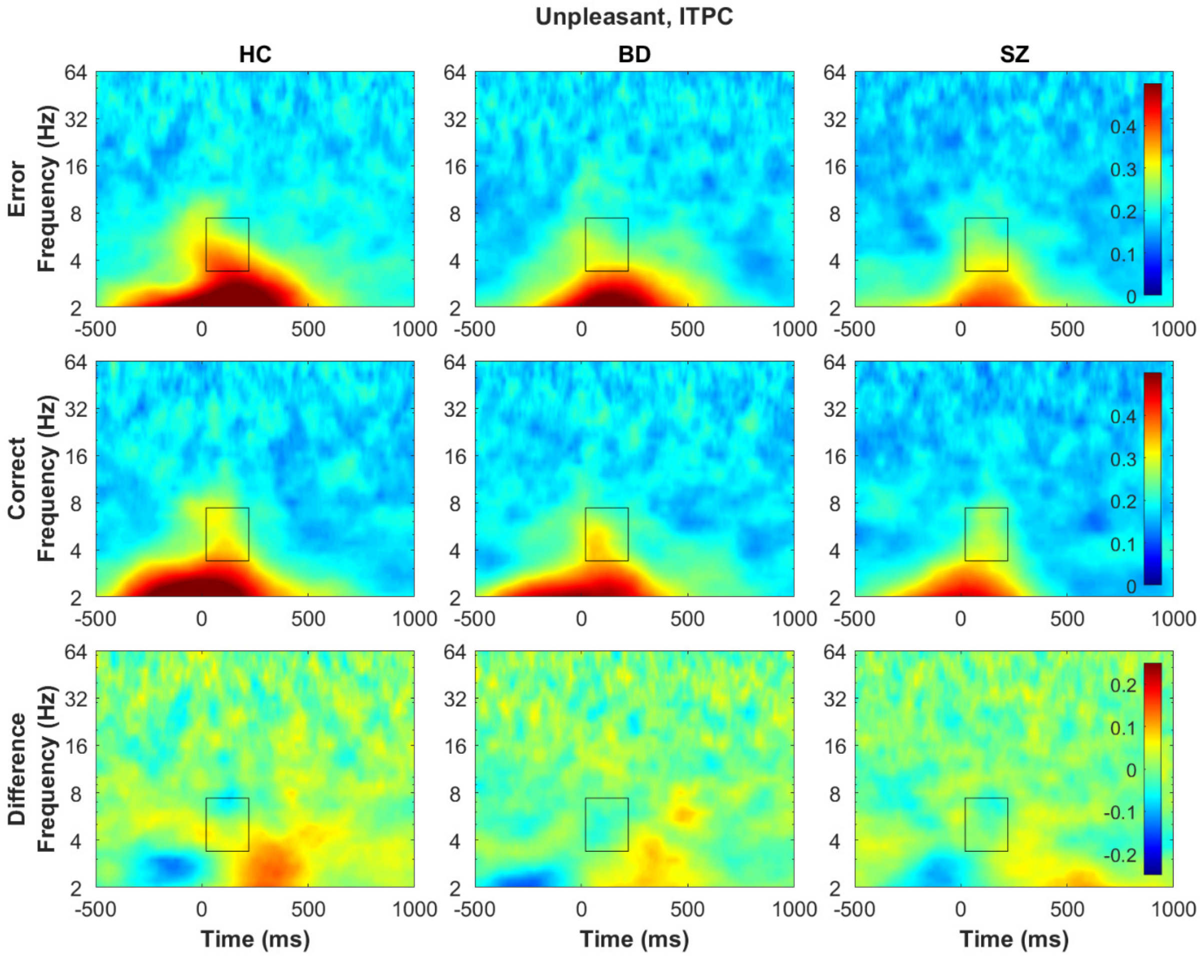

Figure S6. Time-frequency decomposition plots of intertrial phase coherence (ITPC) at Cz electrode for the Unpleasant task. Rows: Top = Error responses; Middle = Correct responses; Bottom = Difference between error and correct responses (error minus correct). Columns: Left = Healthy control group (HC); Middle = Bipolar spectrum disorder group (BD); Right = Schizophrenia spectrum disorder group (SZ). Horizontal axis reflects time (-500ms to 1000ms relative to response; 0ms indicates when response was made) and vertical axis represents the frequency (2 to 64 Hz, logarithmically spaced). Heatmap is scaled for 0 (blue) to 0.5 (red) for the error and correct responses and -0.25 to 0.25 for the difference. The boxed area represents the area that theta-band data was extracted from (20 to 220 ms and 3.38 to 7.38 Hz). Results (Tables 2 & S6): There were no group differences in Error theta ITPC (top row) or Correct theta ITPC (middle row). In all groups, Error theta ITPC was comparable to Correct theta ITPC (bottom row).

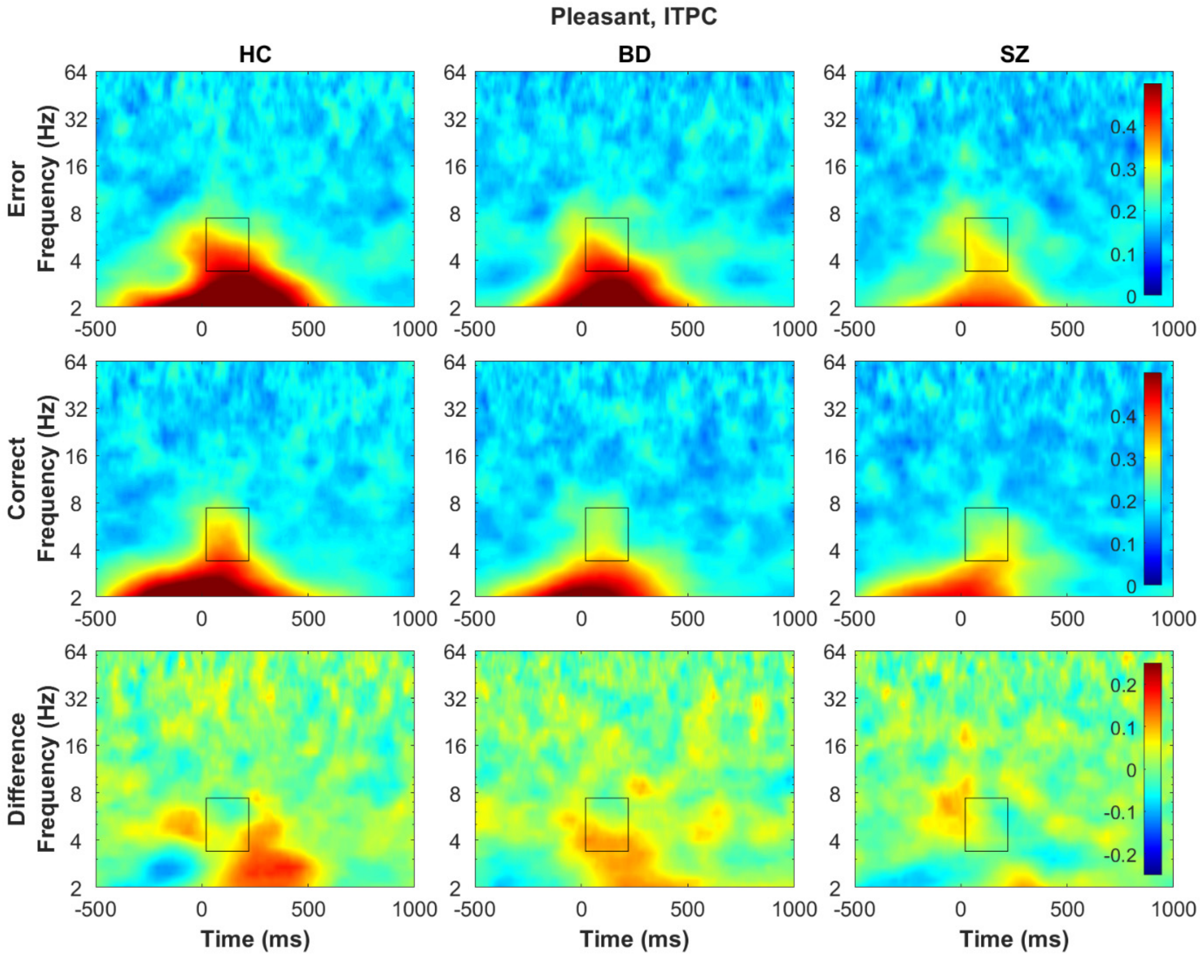

Figure S7. Time-frequency decomposition plots of intertrial phase coherence (ITPC) at Cz electrode for the Pleasant task. Rows: Top = Error responses; Middle = Correct responses; Bottom = Difference between error and correct responses (error minus correct). Columns: Left = Healthy control group (HC); Middle = Bipolar spectrum disorder group (BD); Right = Schizophrenia spectrum disorder group (SZ). Horizontal axis reflects time (-500ms to 1000ms relative to response; 0ms indicates when response was made) and vertical axis represents the frequency (2 to 64 Hz, logarithmically spaced). Heatmap is scaled for 0 (blue) to 0.5 (red) for the error and correct responses and -0.25 to 0.25 for the difference. The boxed area represents the area that theta-band data was extracted from (2 20 to 220 ms and 3.38 to 7.38 Hz). Results (Tables 2 & S6): There were no group differences in Error theta ITPC (top row) or Correct theta ITPC (middle row). In all groups, Error theta ITPC was comparable to Correct theta ITPC (bottom row).

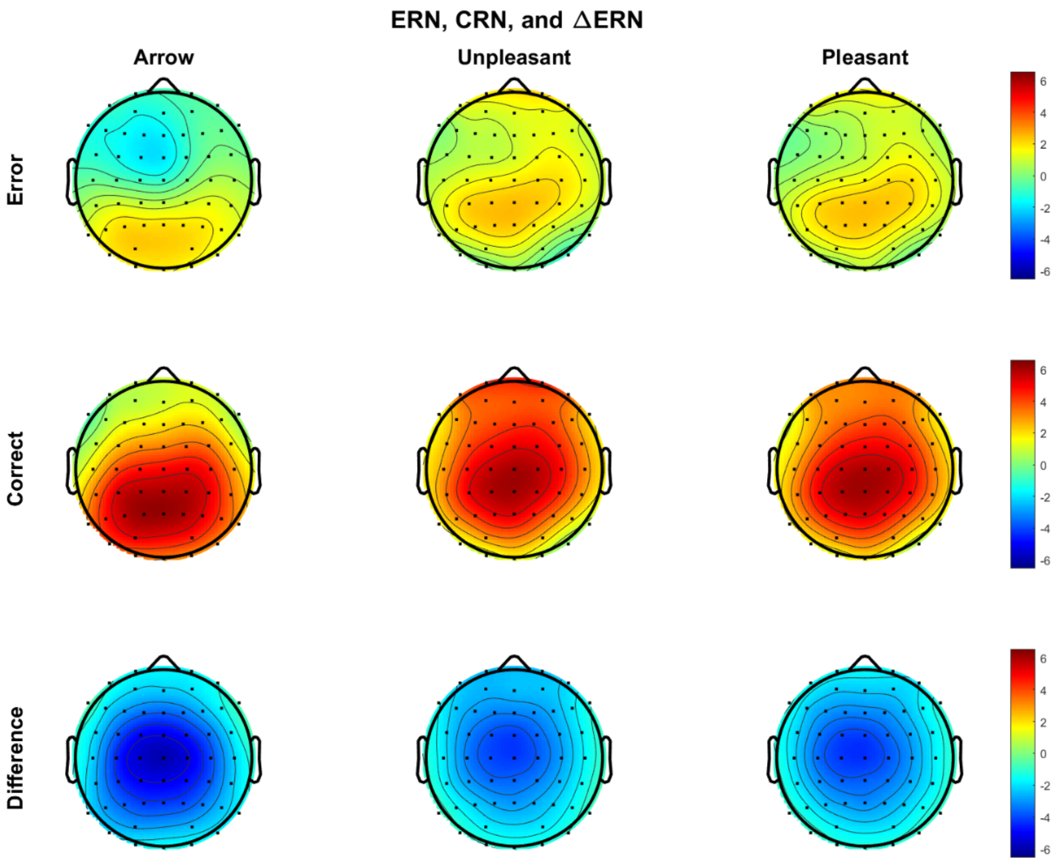

Figure S8. Topography of event-related potentials (ERPs; extracted from -14 to 86 ms), collapsing all three groups (healthy control, bipolar spectrum disorder, schizophrenia spectrum disorder). Rows: Top = Error-related negativity (ERN); Middle = Correct-related negativity (CRN); Bottom = difference between ERN and CRN (ΔERN). Columns: Left = Arrow task; Middle = Unpleasant task; Right = Pleasant task. Heatmap is scaled from -6.2 (blue) to 6.2 (red) microvolts (μV) for all plots for comparability.

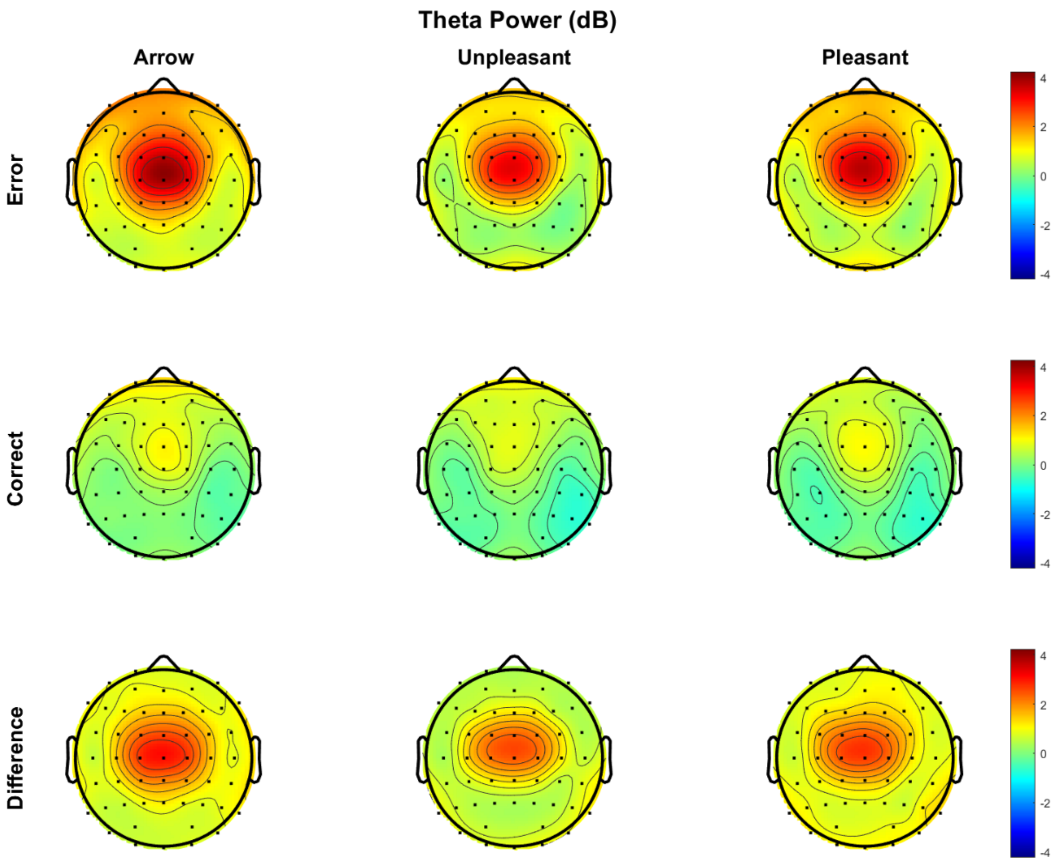

Figure S9. Topography of total power (extracted from 20 to 220 ms and 3.38 to 7.38 Hz), collapsing all three groups (healthy control, bipolar spectrum disorder, schizophrenia spectrum disorder). Rows: Top = Error responses; Middle = Correct responses; Bottom = Difference between error and correct responses (error minus correct). Columns: Left = Arrow task; Middle = Unpleasant task; Right = Pleasant task. Heatmap is scaled from -4.0 (blue) to 4.0 (red) decibel (dB) for all plots for comparability.

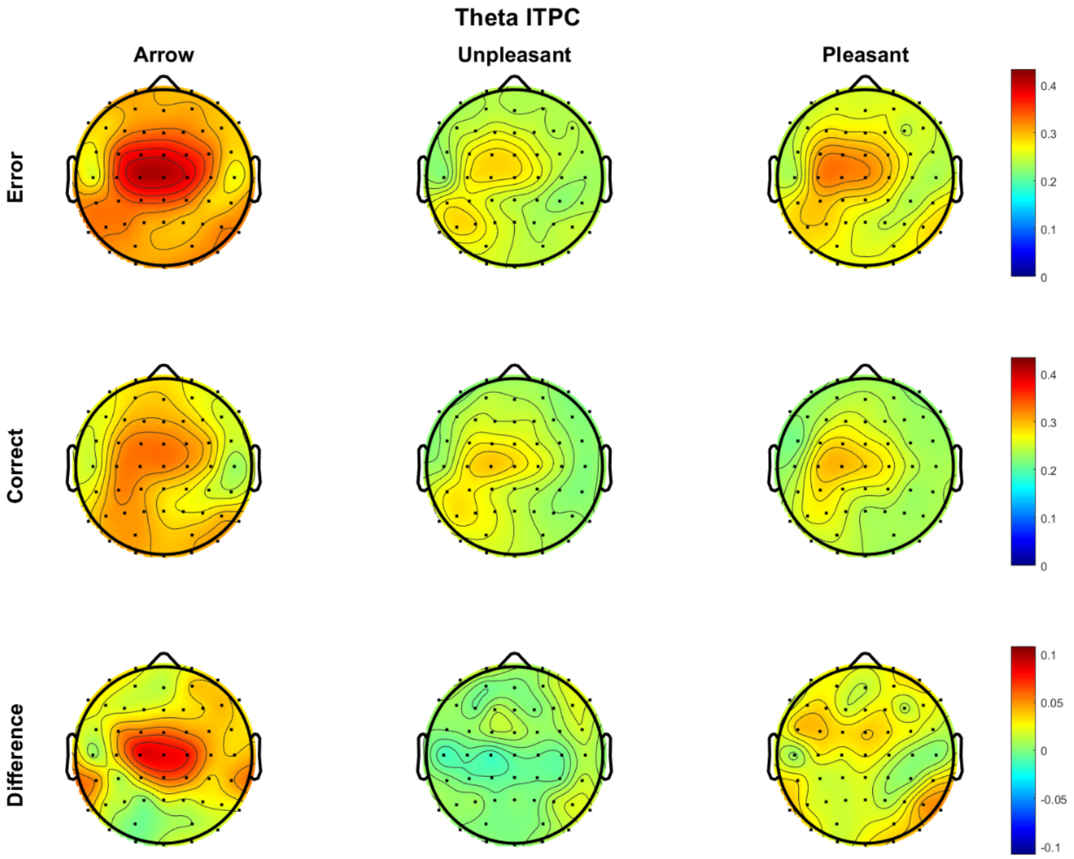

Figure S10. Topography of intertrial phase coherence (ITPC; extracted from 20 to 220 ms and 3.38 to 7.38 Hz), collapsing all three groups (healthy control, bipolar spectrum disorder, schizophrenia spectrum disorder). Rows: Top = Error responses; Middle = Correct responses; Bottom = Difference between error and correct responses (error minus correct). Columns: Left = Arrow task; Middle = Unpleasant task; Right = Pleasant task. Heatmap is scaled from 0 (blue) to 0.41 (red) for the error and correct responses and from -.10 to 0.10 for the difference.

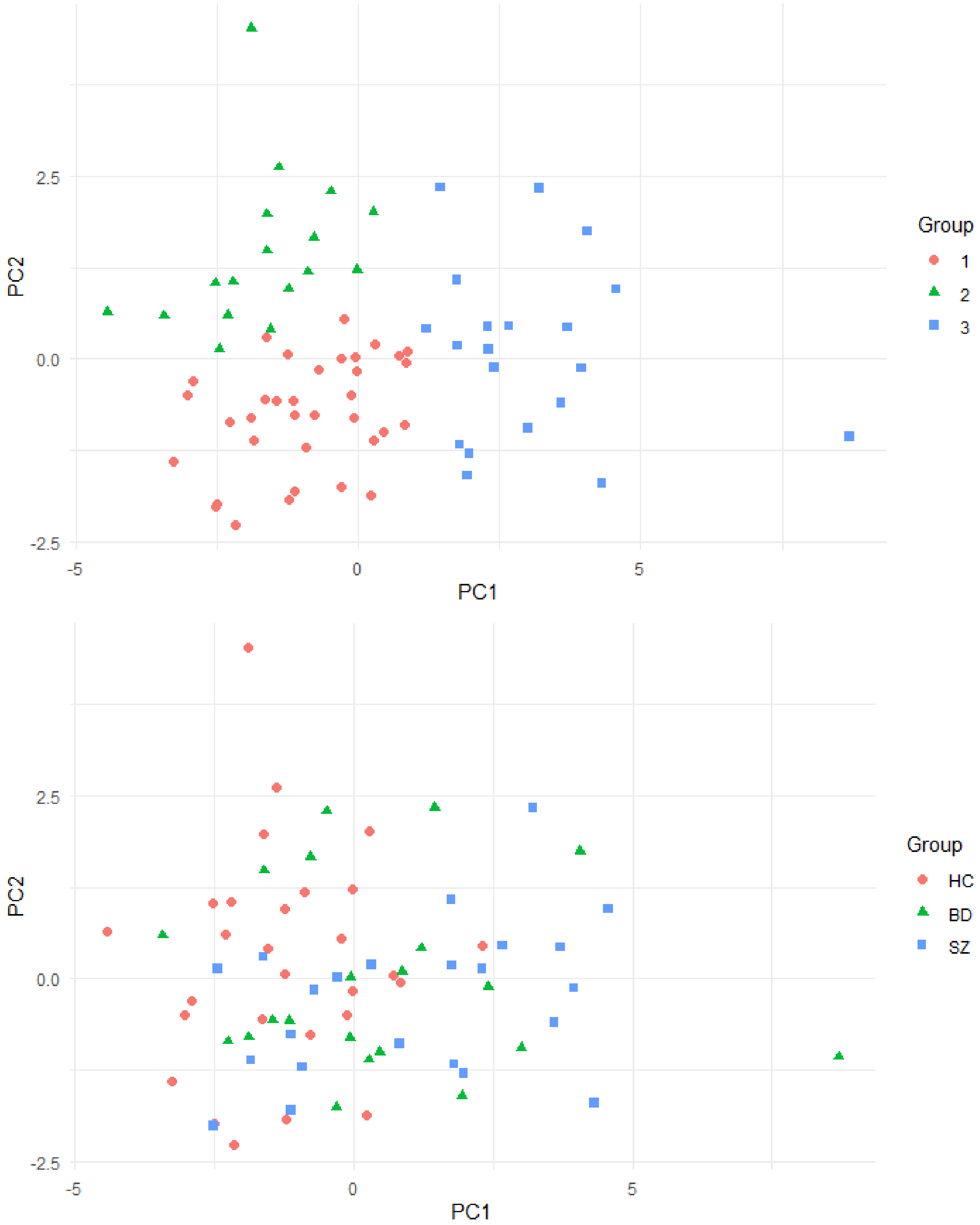

Figure S11. Scatterplots on the first two principal components (PC1 and PC2) by 3-group cluster analysis participants (top; Clusters 1 through 3) and by diagnostic group (bottom; HC = healthy control; BD = bipolar disorder; SZ = schizophrenia).

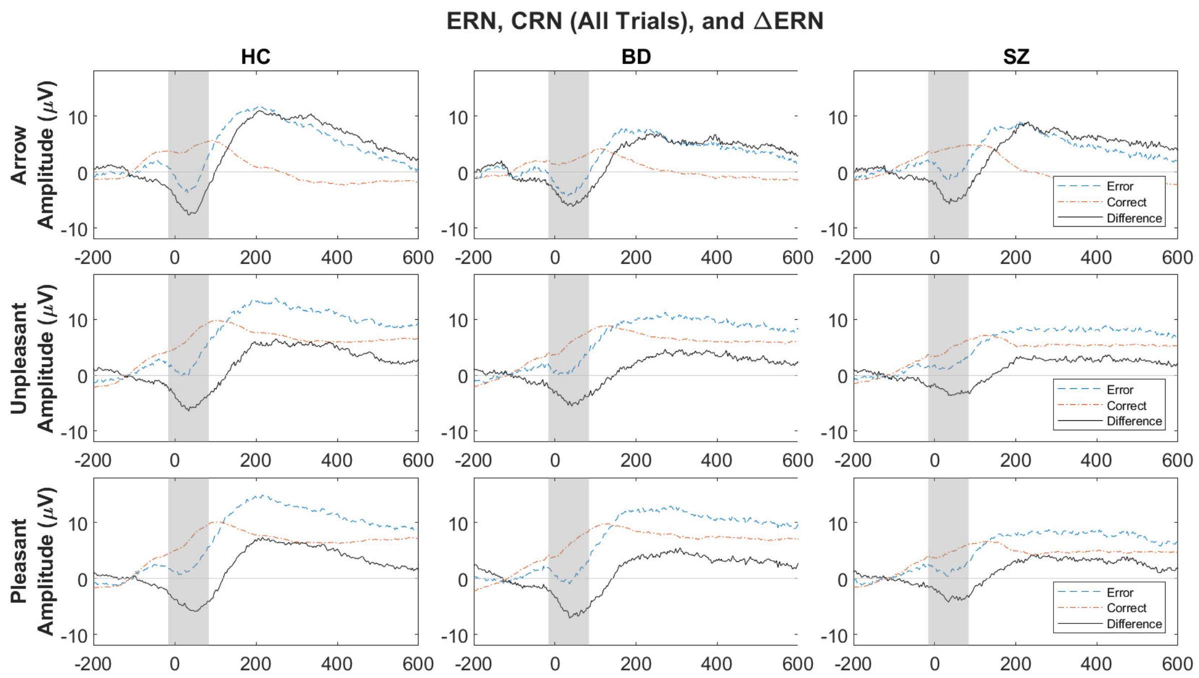

Figure S12. Event-related potential plots at Cz electrode using all trials. Rows: Top = Arrow task; Middle = Unpleasant task; Bottom = Pleasant task. Columns: Left = Healthy control group (HC); Middle = Bipolar spectrum disorder group (BD); Right = Schizophrenia spectrum disorder group (SZ). Blue, dashed lines = Error response; Orange dash-dotted lines = Correct responses; Black, solid line = Difference (Error minus Correct responses). Horizontal axis reflects time (-200ms to 600ms relative to response; 0ms indicates when response was made) and vertical axis represents voltage (microvolts; μV). The gray area represents the area that ERP data was extracted from (-16 to 84 ms). Results (Table 1): In two-way ANCOVAs testing Task effects within each Group, in HC and BD groups, ERPs (ERN and CRN) were larger (more negative) in the Arrow task compared to Unpleasant and Pleasant tasks, while ERPs were comparable across all tasks in SZ. Two-way ANCOVA testing Group effects within each task suggest there were no group differences in ERPs (ERN or CRN; Table 2). Further, there were no interaction effects involving Response, suggesting that ΔERN (ERN minus CRN) were comparable across all groups and tasks.

References

1. Posner K, Brown GK, Stanley B, Brent DA, Yershova KV, Oquendo MA, *et al.* (2011): The Columbia–Suicide Severity Rating Scale: Initial Validity and Internal Consistency Findings From Three Multisite Studies With Adolescents and Adults. *AJP* 168: 1266–1277.
2. First MB, Williams JBW, Karg RS, Spitzer RL (2015): *Structured Clinical Interview for DSM-5—Research Version (SCID-5 for DSM-5, Research Version; SCID-5-RV)*. Arlington, VA, US: American Psychiatric Publishing, Inc.
3. McInnis MG, Assari S, Kamali M, Ryan K, Langenecker SA, Saunders EF, *et al.* (2018): Cohort Profile: The Heinz C. Prechter Longitudinal Study of Bipolar Disorder. *Int J Epidemiol* 47: 28–28n.
4. Yocum AK, Anderau S, Bertram H, Burgess HJ, Cochran AL, Deldin PJ, *et al.* (2023): Cohort Profile Update: The Heinz C. Prechter Longitudinal Study of Bipolar Disorder. *International Journal of Epidemiology* 52: e324–e331.
5. Ryan KA, Vederman AC, McFadden EM, Weldon AL, Kamali M, Langenecker SA, McInnis MG (2012): Differential executive functioning performance by phase of bipolar disorder. *Bipolar Disorders* 14: 527–536.
6. Zimmerman M, Martinez JH, Young D, Chelminski I, Dalrymple K (2013): Severity classification on the Hamilton depression rating scale. *Journal of Affective Disorders* 150: 384–388.
7. Betella A, Verschure PFMJ (2016): The affective slider: A digital self-assessment scale for the measurement of human emotions. *PLOS ONE* 11: e0148037.
8. The Mathworks, Inc. (2024): MATLAB, version 2024a. Natick, MA.

9. Delorme A, Makeig S (2004): EEGLAB: an open source toolbox for analysis of single-trial EEG dynamics including independent component analysis. *Journal of Neuroscience Methods* 134: 9–21.

10. Delorme A (2023): EEG is better left alone [no. 1]. *Sci Rep* 13: 2372.

11. Widmann A, Schröger E, Maess B (2015): Digital filter design for electrophysiological data – a practical approach. *Journal of Neuroscience Methods* 250: 34–46.

12. Tim M (2012): NITRC: CleanLine: Tool/Resource Info. Retrieved September 1, 2020, from https://www.nitrc.org/projects/cleanline

13. Pion-Tonachini L, Kreutz-Delgado K, Makeig S (2019): ICLabel: An automated electroencephalographic independent component classifier, dataset, and website. *NeuroImage* 198: 181–197.

14. Foti D, Kotov R, Hajcak G (2013): Psychometric considerations in using error-related brain activity as a biomarker in psychotic disorders. *Journal of Abnormal Psychology* 122: 520–531.

15. Olvet DM, Hajcak G (2009): The stability of error-related brain activity with increasing trials. *Psychophysiology* 46: 957–961.

16. Boudewyn MA, Luck SJ, Farrens JL, Kappenman ES (2018): How many trials does it take to get a significant ERP effect? It depends. *Psychophysiology* 55: 1–1.

17. Wickham H (2023, October 2): plyr: Tools for Splitting, Applying and Combining Data, version 1.8.9. Retrieved May 11, 2025, from https://cran.r-project.org/web/packages/plyr/index.html

18. Wickham H, François R, Henry L, Müller K, Vaughan D, Software P, PBC (2023, November 17): dplyr: A Grammar of Data Manipulation, version 1.1.4. Retrieved August 23, 2024, from https://cran.r-project.org/web/packages/dplyr/index.html

19. Wickham H (2020, April 9): reshape2: Flexibly Reshape Data: A Reboot of the Reshape Package, version 1.4.4. Retrieved August 23, 2024, from https://cran.r-project.org/web/packages/reshape2/index.html

20. Revelle W (2024, June 27): psych: Procedures for Psychological, Psychometric, and Personality Research, version 2.4.6.26. Retrieved August 23, 2024, from https://cran.r-project.org/web/packages/psych/index.html

21. Bates D, Maechler M, Bolker [aut B, cre, Walker S, Christensen RHB, *et al.* (2024, July 3): lme4: Linear Mixed-Effects Models using "Eigen" and S4, version 1.1-35.5. Retrieved August 23, 2024, from https://cran.r-project.org/web/packages/lme4/index.html

22. Kuznetsova A, Brockhoff PB, Christensen RHB, Jensen SP (2020, October 23): lmerTest: Tests in Linear Mixed Effects Models, version 3.1-3. Retrieved August 30, 2021, from https://CRAN.R-project.org/package=lmerTest

23. Lenth RV, Bolker B, Buerkner P, Giné-Vázquez I, Herve M, Jung M, *et al.* (2024, August 21): emmeans: Estimated Marginal Means, aka Least-Squares Means, version 1.10.4. Retrieved August 23, 2024, from https://cran.r-project.org/web/packages/emmeans/index.html

24. Ben-Shachar MS, Lüdecke D, Makowski D (2020): effectsize: Estimation of Effect Size Indices and Standardized Parameters. *Journal of Open Source Software* 5: 2815.

25. Wickham H, Chang W, Henry L, Pedersen TL, Takahashi K, Wilke C, *et al.* (2024, April 23): ggplot2: Create Elegant Data Visualisations Using the Grammar of Graphics, version

3.5.1. Retrieved August 23, 2024, from https://cran.r-project.org/web/packages/ggplot2/index.html

26. Suzuki T, Ait Oumeziane B, Novak K, Samuel DB, Foti D (2020): Error-monitoring across social and affective processing contexts. *International Journal of Psychophysiology* 150: 37–49.

27. Trujillo LT, Allen JJB (2007): Theta EEG dynamics of the error-related negativity. *Clinical Neurophysiology* 118: 645–668.

28. Meyer A, Weinberg A, Klein DN, Hajcak G (2012): The development of the error-related negativity (ERN) and its relationship with anxiety: Evidence from 8 to 13 year-olds. *Developmental Cognitive Neuroscience* 2: 152–161.

29. Nieuwenhuis S, Ridderinkhof KR, Blom J, Band GPH, Kok A (2001): Error-related brain potentials are differentially related to awareness of response errors: Evidence from an antisaccade task. *Psychophysiology* 38: 752–760.

30. Larson MJ, Clayson PE, Keith CM, Hunt IJ, Hedges DW, Nielsen BL, Call VRA (2016): Cognitive control adjustments in healthy older and younger adults: Conflict adaptation, the error-related negativity (ERN), and evidence of generalized decline with age. *Biological Psychology* 115: 50–63.

31. Klawohn J, Endrass T, Preuss J, Riesel A, Kathmann N (2016): Modulation of hyperactive error signals in obsessive–compulsive disorder by dual-task demands. *Journal of Abnormal Psychology* 125: 292–298.

32. Moser J, Moran T, Schroder H, Donnellan B, Yeung N (2013): On the relationship between anxiety and error monitoring: a meta-analysis and conceptual framework. *Front Hum Neurosci* 7. https://doi.org/10.3389/fnhum.2013.00466

33. Luck SJ, Stewart AX, Simmons AM, Rhemtulla M (2021): Standardized measurement error: A universal metric of data quality for averaged event-related potentials. *Psychophysiology* 58: 1–15.
